# Comparative Genome-Wide-Association Mapping Identifies Common Loci Controlling Root System Architecture and Resistance to *Aphanomyces euteiches* in Pea

**DOI:** 10.3389/fpls.2017.02195

**Published:** 2018-01-05

**Authors:** Aurore Desgroux, Valentin N. Baudais, Véronique Aubert, Gwenola Le Roy, Henri de Larambergue, Henri Miteul, Grégoire Aubert, Gilles Boutet, Gérard Duc, Alain Baranger, Judith Burstin, Maria Manzanares-Dauleux, Marie-Laure Pilet-Nayel, Virginie Bourion

**Affiliations:** ^1^Institut de Génétique, Environnement et Protection des Plantes, INRA, Agrocampus Ouest, Université Rennes 1, Le Rheu, France; ^2^Agroécologie, INRA, AgroSup Dijon, Université Bourgogne Franche-Comté, Dijon, France; ^3^PISOM, UMT INRA/Terre Inovia, Le Rheu, France

**Keywords:** root system architecture, disease resistance, *Pisum sativum*, GWAS, root rot

## Abstract

Combining plant genetic resistance with architectural traits that are unfavorable to disease development is a promising strategy for reducing epidemics. However, few studies have identified root system architecture (RSA) traits with the potential to limit root disease development. Pea is a major cultivated legume worldwide and has a wide level of natural genetic variability for plant architecture. The root pathogen *Aphanomyces euteiches* is a major limiting factor of pea crop yield. This study aimed to increase the knowledge on the diversity of loci and candidate genes controlling RSA traits in pea and identify RSA genetic loci associated with resistance to *A. euteiches* which could be combined with resistance QTL in breeding. A comparative genome wide association (GWA) study of plant architecture and resistance to *A. euteiches* was conducted at the young plant stage in a collection of 266 pea lines contrasted for both traits. The collection was genotyped using 14,157 SNP markers from recent pea genomic resources. It was phenotyped for ten root, shoot and overall plant architecture traits, as well as three disease resistance traits in controlled conditions, using image analysis. We identified a total of 75 short-size genomic intervals significantly associated with plant architecture and overlapping with 46 previously detected QTL. The major consistent intervals included plant shoot architecture or flowering genes (*PsLE, PsTFL1*) with putative pleiotropic effects on root architecture. A total of 11 genomic intervals were significantly associated with resistance to *A. euteiches* confirming several consistent previously identified major QTL. One significant SNP, mapped to the major QTL *Ae-Ps7.6*, was associated with both resistance and RSA traits. At this marker, the resistance-enhancing allele was associated with an increased total root projected area, in accordance with the correlation observed between resistance and larger root systems in the collection. Seven additional intervals associated with plant architecture overlapped with GWA intervals previously identified for resistance to *A. euteiches*. This study provides innovative results about genetic interdependency of root disease resistance and RSA inheritance. It identifies pea lines, QTL, closely-linked markers and candidate genes for marker-assisted-selection of RSA loci to reduce Aphanomyces root rot severity in future pea varieties.

## Introduction

Plant architecture has often been reported to play a role in modifying organ susceptibility to pathogens or pests, by favoring mechanisms leading to infection escape or increased tolerance (Ney et al., 2013). Spatial disease avoidance was shown to result from a combination of architectural features with unfavorable effects on disease development and severity (Tivoli et al., 2013). Combining plant genetic resistance with the architectural traits that are the most unfavorable to diseases would thus be a strategy of interest for reducing epidemics. Most reports of exploiting plant architecture effects to limit disease development were carried out on the aerial parts of the plant. In one successful example, cultivars were bred for an upright growth habit, lodging-resistance, and partial intrinsic resistance which improved the management of white mold in bean, soybean, canola, peanut, and potato (McDonald et al., 2013). In contrast, few studies have identified and used root architecture traits in breeding to limit root disease development, probably due to the difficulty in evaluating the effects of both disease and plant architecture on the root compartment (Downie et al., 2015).

Plant roots are crucial for water and nutrient supply, as well as for anchorage into the soil. The spatial configuration of the root system, the so-called “root system architecture” (RSA), varies greatly according to both intrinsic and environmental determinants (Malamy, 2005). Intrinsic determinants are essential for organogenesis and growth, and determine the RSA characteristics in a given plant species. Environmental determinants are numerous and include the soil nutritional composition, density and compaction, salinity and water content, and the presence of micro-organisms. Plant RSA is the result of growth and developmental processes. The root system originates from a primary root that develops during embryogenesis. This primary root produces secondary roots, which in turn produce tertiary roots. All secondary, tertiary, quaternary, and further level roots are referred to as lateral roots. The RSA is generally characterized by measuring variables such as secondary lateral root number, root length, and average diameter. In contrast, variables describing the variety of components constituting the relationship between the root segments (e.g. type and angle of connexion between roots; root gradients) refer to the root system topology or structure (Hodge et al., 2009). The intrinsic determinants of RSA are those which are essential for developmental patterning of the primordium, lateral root initiation, and lateral root emergence and elongation (Malamy, 2005). In response to environmental stimuli, plants can optimize their RSA by both initiating more or less lateral root primordia and influencing growth of primary or lateral roots. Thus, RSA results from the expression of numerous quantitative traits mainly controlled by a large number of loci (Hodge et al., 2009). A few studies have shown the influence of some RSA traits on disease severity due to soil-borne pathogens. A large number of lateral roots, high root diameter, or root dry weight were shown to be correlated with Fusarium root rot resistance in legume plants such as common bean (Snapp et al., 2003; Román-Avilés et al., 2004; Cichy et al., 2007; Hagerty et al., 2015) and pea (Kraft and Boge, 2001).

Dry pea (*Pisum sativum*) is one of the most cultivated grain legumes throughout the world, used both as animal feed and human food. Its symbiotic relationship with *Rhizobium* to capture atmospheric dinitrogen makes it a valuable crop in rotations that allows decreasing chemical nitrogen use. *Aphanomyces euteiches*, a soilborne pathogen that infects the roots of different legume hosts, is a limiting factor in pea crop development since it can cause high yield losses in infested fields (Gaulin et al., 2007). Under favorable weather conditions (temperatures above 10°C and very wet soil), the disease causes damaging browning of roots on young plants. Genetic resistance to *A. euteiches* in pea has been well-explored in the last decade and has been shown to be partial and controlled by numerous Quantitative Trait Loci (QTL) (Pilet-Nayel et al., 2002, 2005; Hamon et al., 2011, 2013; Desgroux et al., 2016). However, the potential of plant RSA to limit Aphanomyces root rot symptoms has not yet been thoroughly investigated in pea. McPhee (2005) identified the pea Aphanomyces root rot partially resistant genotype *PI180693* among the pea accessions with the highest root:aerial dry weight ratio out of 330 accessions evaluated for seedling RSA traits. In *Medicago truncatula*, resistance to *A. euteiches* was also associated with a high number of lateral roots in several genotypes (Djébali et al., 2013; Bonhomme et al., 2014; Laffont et al., 2015). Pea has a large genetic variability for RSA traits between genotypes, from seedling stage to mature plant (McPhee, 2005; Bourion et al., 2010). Pea root architecture has been reported to be under polygenic control. Three, eight and 21 QTL were identified for root dry matter, number of lateral roots and root length, respectively, in a recombinant inbred line (RIL) population (Bourion et al., 2010). However, little is known about the diversity of genetic determinants of root architecture in pea natural variability.

The present study aimed to (i) improve current knowledge on the diversity of QTL and candidate genes controlling RSA traits in pea and (ii) compare the genomic localization of loci controlling RSA traits and Aphanomyces root rot resistance at the young plant stage, and (iii) identify common RSA and resistance loci that would be useful in breeding. A genome wide association (GWA) study was carried out to benefit from the advantages of the precise and multiple allele detection permitted by this approach (Gupta et al., 2014). A pea collection of 266 accessions, combining a wide range of phenotypes for RSA traits and resistance to *A. euteiches*, was used. The collection was genotyped using 14,157 recent SNP (Single Nucleotide Polymorphism) resources developed in pea (Tayeh et al., 2015; Boutet et al., 2016). The collection was phenotyped for diverse plant architecture traits, especially in the root system, and for resistance to *A. euteiches* in young plants under controlled conditions. Correlation analysis between RSA and resistance data identified relationships between plant architecture and resistance traits. The GWA study confirmed the diversity of genomic regions associated with plant architecture, and identified common loci associated with RSA traits and resistance to *A. euteiches*.

## Materials and methods

### Plant material

A collection of 266 pea lines, including the 175 lines of the pea-Aphanomyces collection previously described in Desgroux et al. (2016) and the 104 lines of the pea core-collection previously presented in Bourion et al. (2018), was used in this study. There were 13 in common between the pea-Aphanomyces collection and the pea core-collection. The pea-Aphanomyces collection is representative of the genetic and phenotypic variability identified or created in pea for resistance vs. susceptibility to *A. euteiches*. It includes: (i) 58% resistant or susceptible lines derived from a French recurrent-selection based breeding program, (ii) 28% partially resistant pea RILs or wild and germplasm lines derived from INRA and USDA Aphanomyces research programs, and (iii) 14% susceptible spring or winter pea varieties grown in Europe. The pea core-collection is representative of the genetic or biogeographic diversity and the variability in agronomic traits found within the genus *Pisum*. The 104 lines originate from 36 countries located in *Pisum* centers of diversity and domestication or in areas where domesticated peas were disseminated, and consist of wild or semi-wild genotypes, landraces, inbred lines or germplasm, and cultivars.

### Phenotyping

All 266 lines were assayed in three experiments, including one experiment at INRA-Dijon, Burgundy, France (Exp#1) and two experiments at INRA-Le Rheu, Brittany, France (Exp#2 and Exp#3).

Exp#1 was conducted in a greenhouse, at 23°C for 16 h-day and 18°C for 8 h-night. All the lines were assayed in a randomized complete block design with two replicates. Four surface sterilized seeds per replicate were sown in a pot (12 × 12 × 20 cm) for each pea line. Plants were grown in a 1:1 (v/v) mixture of sterilized attapulgite and clay balls (2–6 mm diameter; Bourion et al., 2010) and watered as needed. In each pot, two eight-day old seedlings were carefully uprooted, washed and stored at 5°C before plant architecture measurements.

Exp#2 was carried out in a climate controlled chamber at 25°C for 16 h-day and 23°C for 8 h-night. All the lines were assayed in a randomized complete block design with three replicates. Five seeds per replicate were sown in a pot (9 × 9 × 9.5 cm) for each pea line. Plants were grown in vermiculite substrate and watered as needed. Fourteen-day old seedlings were uprooted and two seedlings per pot were carefully washed before plant architecture trait measurements.

In Exp#1 and Exp#2, different plant traits related to both growth and architecture were measured or calculated as described in Bourion et al. (2010). Tap root length (TapRootL), number of first lateral roots (NLatRoot), and plant height (ShootL) were first measured on each plant. Then, the root and aerial parts were separated at the cotyledon insertion point; roots were carefully spread on a transparent sheet and scanned with a blue background at 300 dpi (Exp#1: A3 color scanner, Epson, Tokyo, Japan; Exp#2: A4 color scanner, Epson Perfection V37 J232C, Tokyo, Japan). Images were then analyzed using Winrhizo® Software (Regent Instruments, Quebec, Canada) with a home-made color scale to take into account the maximum root area. Several traits were scored from image analyses: total root projected area (TProjArea), total root length (TRootL), and average root diameter (RootDia). Average lateral root length (LatRootL) was computed from measurements and image analyses as following: LatRootL = (TRootL − TapRootL)/NLatRoot. Roots and shoots were then dried at 80°C for 48 h and weighed to obtain root biomass (RootB) and shoot biomass (ShootB). Total biomass (TB) and root to total biomass ratio (RootB:TB) were computed as TB = RootB + ShootB and RootB:TB = RootB/TB. Six of those traits, NLatRoot, TRootL, LatRootL, TProjArea, RootDia, and RootB, were used in this study as RSA traits, two others, ShootL and ShootB, as shoot architecture traits, and the last two, TB and RootB:TB, as overall plant architecture traits.

**Figure 1:**
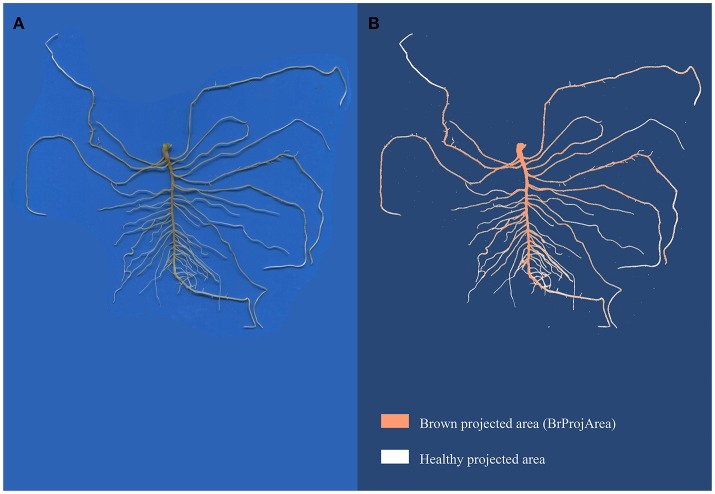
Image acquisition of a root system **(A)** and image analysis with Winrhizo® **(B)**. Root images were acquired with a color scanner at 300 dpi. The images were then analyzed with Winrhizo® software using a homemade color scale to discriminate brown and white parts of roots.

Exp#3 was carried out in the same climatic chamber as Exp#2, at 25°C for 16 h-day and 23°C for 8 h-night, as described in Desgroux et al. (2016). Seven-day old seedlings were inoculated with a pure-culture of RB84, the French reference strain of *A. euteiches* described in Hamon et al. (2011), referred to as pathotype I (Wicker and Rouxel, 2001). Inoculation was performed with a solution of 10^3^ zoospores per plant, produced as previously described (Moussart et al., 2001). Seven days after inoculation, disease severity (DS) was assessed on five plants per plot, using a 0 (no symptoms) to 5 (dead plant) scoring scale proportional to the percentage of browning symptoms on roots and epicotyls (Hamon et al., 2011). For each genotype, a Root Rot Index (RRI) was then calculated as the mean disease severity score of the five plants in a pot. Among the five plants, two were chosen for a complementary description of the damage on roots from image analyzes using image analysis. Their roots were carefully spread on a transparent sheet and scanned as described for Exp#1 and Exp#2. Image analyses were then performed using the Winrhizo® Software with a home-made color scale which enabled to discriminate between healthy and diseased root projected area. A percentage of root system with browning symptoms (Br:TProjArea) was calculated as Br:TProjArea = BrProjArea × 100/TProjArea, with BrProjArea, the projected area detected as brown by Winrhizo®, and TProjArea, the total projected area (Figure 1).

### Statistical analysis of phenotypic data

Phenotypic datasets for plant architecture traits and resistance to *A. euteiches*, obtained from the 266-pea collection were analyzed using the R 3.1.1 program (R Core Team, 2014). For each variable, a linear model (LM) [R function lm] was used, including G (genotype), R (replicate) and P (plant) as fixed factors. Normality of residuals and homogeneity of their variance were checked using Skewness, Kurtosis and Shapiro-Wilk statistics (*p-value* ≥ 0.05), as well as Bartlett test (*p-value* ≥ 0.05), as described in Desgroux et al. (2016). Mean-based heritability (h^2^) was calculated for each variable from variance estimates using the formula h^2^ = σ_G_^2^ / [σ_G_^2^ + (σ_E_^2^/*r*)], where σ_G_^2^ is the genetic variance, σ_E_^2^ the residual variance and *r* the number of replicates per line. Least Square Means (LSMeans) were calculated from each LM analysis (R function lsmeans of package lsmeans; Lenth and Hervé, 2015). Histograms of LSMeans frequency distributions were drawn using the R function hist. Pearson correlation analysis was carried out between LSMeans of the different variables (R function corr.test of package psych; Revelle, 2015). Significance of the Pearson correlations was tested with a false discovery rate correction for multiple testing (corrected *p-value* < 0.05; Benjamini and Hochberg, 1995). A regression curve was drawn between the Br:TProjArea and DS data using the linear model Br:TProjArea~DS.

### Genetic analysis

#### Genotyping and consensus map

The pea lines were genotyped using a total of 14,157 SNP markers, including the 13,204 SNP markers from the GenoPea Infinium SNP Array (Tayeh et al., 2015) and 953 SNP markers from Boutet et al. (2016). The 953 SNPs were chosen to cover QTL regions previously found to be associated with disease resistance, especially Aphanomyces root rot (Hamon et al., 2013). Genotyping data with the 13,204 SNPs were obtained previously, as described in Desgroux et al. (2016) and Bourion et al. (2018). Genotyping data with the 953 SNPs were obtained in this study from the same DNA samples as for the Infinium assay, using KASP™ SNP assays carried out in LGC Genomics service lab, UK (http://www.lgcgenomics.com) as described in Boutet et al. (2016). Each line was coded “AA” or “BB” when homozygous for the first or second allele and “AB” when heterozygous.

The genotyping dataset of the pea collection was reduced to 12,812 SNP markers based on genotyping quality, and was filtered using PLINK 1.9 software (Purcell et al., 2007; Chang et al., 2015). Six of the 266 pea lines, i.e., the same lines as in Desgroux et al. (2016), with missing data for more than 10% of SNP markers were excluded from the GWA analysis. Markers with missing data that exceeded 10% or with a minor allele frequency (MAF) lower than 5% in the 260 remaining lines, were also removed from the analysis. A total of 11,789 SNP markers were thus retained for the genetic analysis.

The genotyping raw data set of 11,789 SNP markers, containing 0.7% missing values, was imputed using the R function knncatimputeLarge (package scrime; Schwender and Fritsch, 2013). Imputation parameters were tested with 10 replicates, using a subset of 4,805 SNP markers of the dataset with no missing values. For each replicate, 0.7% missing values were randomly simulated and imputation parameters were tested for one to 50 nearest neighbors and four different methods to determine distances between SNPs, as described in Desgroux et al. (2016). Parameters with the lower error rate over the 10 replicates (eight nearest neighbors and Cohen's kappa method; error rate: 17.06%) were applied to the 11,789-SNP-marker dataset to impute missing values.

The two marker sets used in this study, derived from the GenoPea Infinium SNP Array and the KASP™ assays, respectively, were previously mapped to different genetic maps (Tayeh et al., 2015; Boutet et al., 2016; Desgroux et al., 2016). In this study, a consensus marker map was obtained by projecting the 953 SNP positions from the 64,263 markers map of Boutet et al. (2016) (namely BP-WGGBS map), onto the consensus map established by Desgroux et al. (2016) (namely THMap). For this the “iterative map projection” tool of Biomercator V4.2 software was used (Sosnowski et al., 2012). The THMap contained the GenoPea Infinium array-SNPs, as well as individual- and meta-QTL previously identified for Aphanomyces resistance from linkage (Hamon et al., 2011, 2013) or association (Desgroux et al., 2016) mapping. Seventy-eight markers were removed from the new consensus marker map because they were located within inversions. The level of connectivity between the consensus marker map created and the THMap was estimated with the “InfoMap” tool of the software.

Pairwise Linkage Disequilibrium (LD) between markers was explored within Linkage Groups (LGs) from imputed genotypic data using PLINK 1.9 software. The square correlation coefficient (*r*^2^) values obtained were then plotted against genetic distances (cM), according to the consensus marker map obtained in this study, to estimate the LD decay. LD decay curve and rate for each LG were estimated as described in Desgroux et al. (2016), based on the consensus genetic map from Tayeh et al. (2015).

#### Population structure, individual relatedness and genome-wide association

To estimate the structure of the collection, a Principal Component Analysis (PCA) and a Kinship relatedness matrix were carried out, using the SNP marker dataset and the EMMA (efficient mixed-model association) method in the GAPIT (Genome Association and Prediction Integrated Tool) R package (Lipka et al., 2012) as reported in Desgroux et al. (2016).

GWA analyses used (i) LSMean phenotypic data for six RSA traits (NLatRoot, TRootL, LatRootL, TProjArea, RootDia, RootB), two shoot architecture traits (ShootL, ShootB), two overall plant architecture traits (TB and RootB:TB) and three Aphanomyces root rot resistance variables (RRI, DS and Br:TProjArea) and (ii) genotyping data at 11,789 SNP markers over all the seven LGs. GWA analyses were performed using a modified version of the multi-locus mixed model (MLMM) R package (Segura et al., 2012), as described in Desgroux et al. (2016). The PCA matrix of population structure and the Kinship matrix were defined as cofactors in the MLMM (see the mlmm_cof.r R script at https://sites.google.com/site/vincentosegura/mlmm). Significant SNP markers were also used as cofactors in a forward/backward regression model. A multiple-Bonferroni (mBonf) threshold of 4.58 (*p-value* of 2.6E-5), taking into account 3,840 distinct genetic positions on the created consensus marker map, was used to declare significant SNPs. In each GWA analysis, the optimal MLMM step was determined as the largest stepwise mixed model regression in which all cofactors have −log (*p-value*) above the mBonf threshold defined. Local LD analysis was used to define the confidence intervals (CIs) around significant associated markers detected by GWA, using Plink 1.9 software. Each CI was determined as the interval containing markers in LD (*r*^2^ > 0.2) with the significant associated marker, as previously described (Desgroux et al., 2016; Pascual et al., 2016).

#### Comparative mapping

The genetic map described in Bourion et al. (2010) was previously used to map QTL for root and aerial architecture traits in pea (Bourion et al., 2010) and shares 141 common markers with the consensus marker map developed in this study. Seven RSA and aerial architecture traits analyzed in the previous study were common to the present one, including ShootL, ShootB, TB, TRootL, NLatRoot, RootB, and RootB:TB, this last trait being similar to the below ground to total biomass ratio (BGB:TB) measured on young plants without nodules in Bourion et al. (2010). Plant architecture QTL detected in Bourion et al. (2010) were thus projected onto the present consensus marker map, using Biomercator V4.2 software as described in Desgroux et al. (2016).

The loci detected by association and linkage mapping were visualized on the resulting comparative marker map using MapChart 2.1 software (Voorrips, 2002).

## Results

### Analysis of phenotypic data

Statistical analysis of plant architecture variables, obtained on the 266-pea-line collection at 8 and 14 days after sowing in Exp#1 and Exp#2, respectively, revealed highly significant G effects (*p-value* < 0.001) for all the variables (Supplementary Table 1). Heritability of root, shoot and overall plant architecture traits was higher than 0.7 in both Exp#1 and Exp#2. Frequency distribution of for each variable tended to fit normal curves (Supplementary Figure 1), except for ShootL which showed bimodal distribution in Exp#2. Statistical analysis of the disease variables RRI, DS and Br:TProjArea, obtained in Exp#3, revealed highly significant G effects (*p-value* < 0.001) and high heritability values (h^2^ > 0.82) (Supplementary Table 1). Frequency distribution of the disease variables showed a large range of variation in disease severity in the collection, with resistant and susceptible lines (Supplementary Figure 1).

Most of the RSA variables measured in Exp#1 or Exp#2, except RootDia, were highly significantly and positively correlated between each other (Table 1). Indeed, RootDia were mostly significantly and negatively correlated with other RSA variables, except RootB. In both experiments, shoot architecture variables were significantly and positively correlated with most RSA variables, except RootDia. The correlations between overall plant architecture and RSA or shoot architecture variables were mostly significant and positive, except those between the root to total biomass ratio (RootB:TB) and shoot architecture variables which were significantly negative. Lastly, each variable in Exp#1 was significantly and positively correlated with the same variable in Exp#2.

**Table 1:**
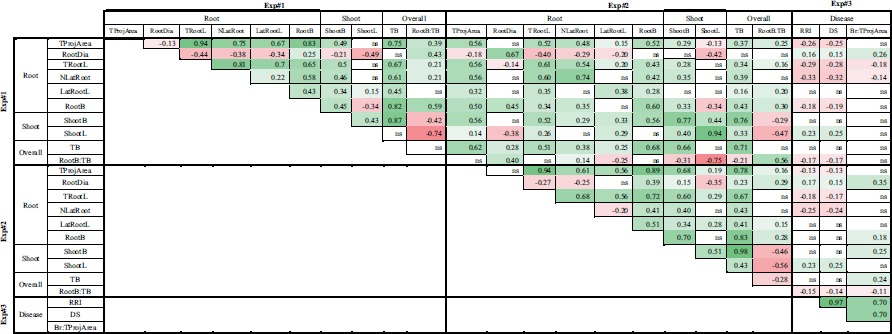
Pearson correlation coefficients between variables collected in three experiments (Exp#1, Exp#2, Exp#3).

*Architecture traits were assessed on 8 day-old plants in a greenhouse (Exp#1) and 14 day-old plants in climate controlled chambers (Exp#2). Disease traits were assessed on 14 day-old plants inoculated at the 7th day with a reference strain of A. euteiches (RB84) in a climatic chamber (Exp#3). Traits are coded as followed: Root architecture traits: TProjArea, total root projected area; RootDia, average root diameter; TRootL, total root length; NLatRoot, number of lateral roots; LatRootL, average length of lateral roots; RootB, root biomass; Shoot architecture traits: ShootB, shoot biomass; ShootL, shoot length; Overall architecture traits: TB, total biomass; RootB: TB, root to total biomass ratio; Disease traits: RRI, Root rot index; DS, disease severity score; Br: TProjArea, percentage of brown projected area. Only correlations with a p < 0.05 are indicated. ns, non–significant correlation (p > 0.05). Level of correlation is coded with a color gradation scale from dark green or red (r = +/−1) to white (r = 0)*.

In Exp#3, the two disease variables, RRI and DS, were significantly and positively correlated with Br:TProjArea (Table 1). Accordingly, the regression curve of Br:TProjArea LSMeans plotted against DS LSMeans showed a coefficient of determination of 0.48 (Supplementary Figure 2). RRI, DS and Br:TProjArea were significantly correlated with most architecture traits measured in Exp#1 and Exp#2. They were negatively correlated with most of the RSA variables (TProjArea, TRootL, NLatRoot, RootB:TB) and positively correlated with RootDia and ShootL (Table 1). Indeed, several pea lines, including *AeD99OSW-58-10-5, AeD99OSW-50-2-5, AeD99OSW-47-6-1, AeD99OSW51-2-10*, RIL 846-07, and *AeD99QU-04-4-6-1*, had both a low disease severity score (RRI < 2.3), high number of lateral roots (NLatRoot > 50) and high total root length (TRootL > 400 cm). In contrast, almost no correlation was observed between disease traits and LatRootL, ShootB or TB.

### Genetic analysis

#### Linkage disequilibrium, structure, and kinship

The LD decay was estimated from the imputed genotyping data of the collection obtained with the filtered 11,789 SNP markers, and ranged from 0.034 to 0.115 cM, depending on the LG (Supplementary Figure 3). The average value was 0.055 over all the LGs, which is lower than the mean LD decay value (0.12 cM) estimated by Desgroux et al. (2016) on 175 of the 266 pea lines in the collection.

**Figure 2:**
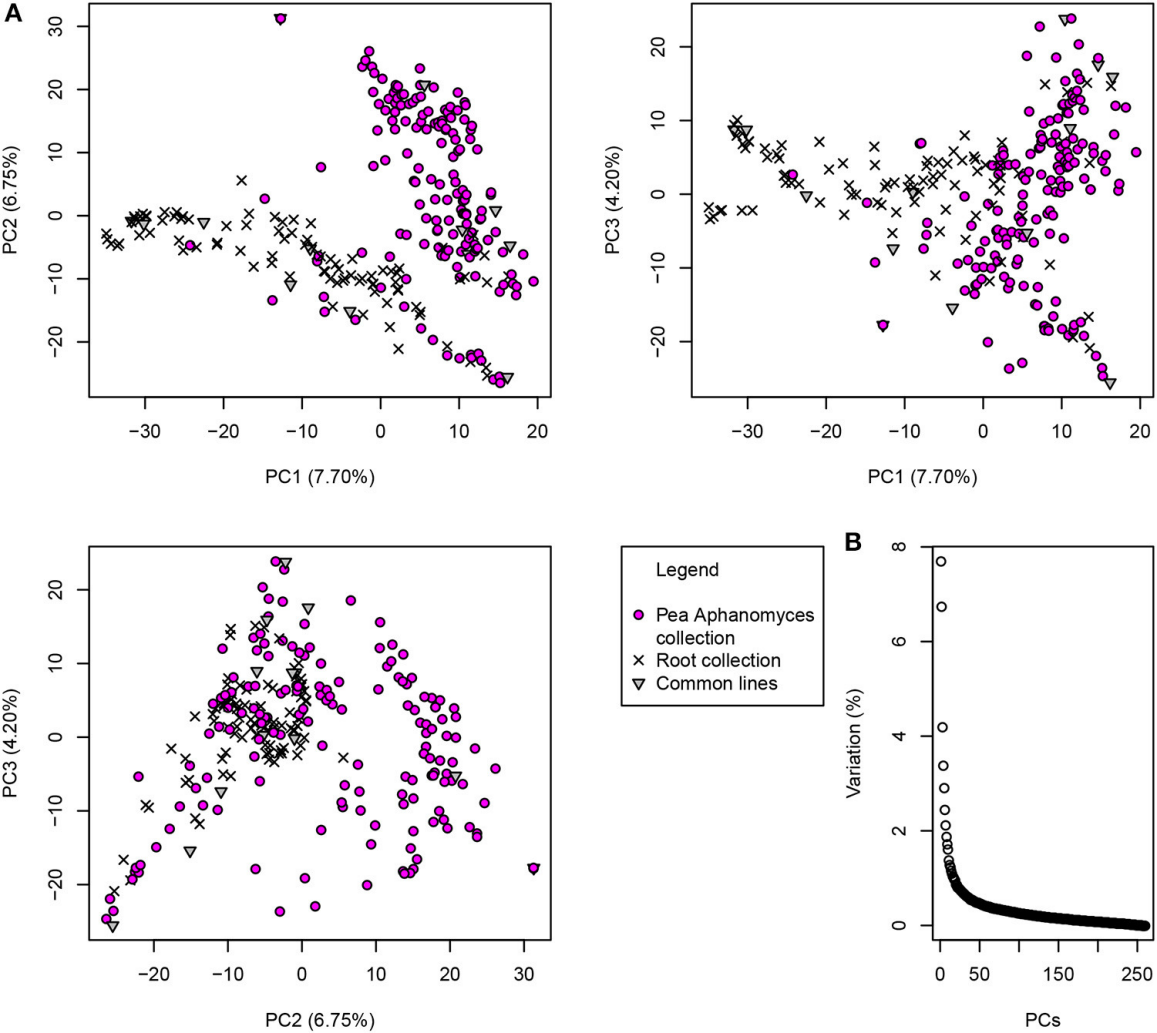
Population structure of the 266-pea-line collection based on the two first axes of the Principal Component Analysis (PCA). PCA was carried out with the GAPIT R package (Lipka et al., 2012), based on 2,937 markers mapped to distinct genetic positions according to Tayeh et al. (2015). **(A)** Lines from the 266-pea-line collection are represented on the first three principal components that explain a total of 18.65% of inertia. Pea-Aphanomyces collection: 162 pea lines specific to the pea-Aphanomyces collection described in Desgroux et al. (2016); Pea core-collection: 91 pea lines specific to the pea core-collection described in Bourion et al. (2018); Common lines: 13 common lines belonging to both the pea-Aphanomyces collection and the pea core-collection. **(B)** Inertia contribution of each principal component (from PC1 to PC260).

The first three principal components of the PCA analysis explained a total of 18.6% of the genetic variation in the 266-pea-line collection. The first PC contributed up to 7.70% of the variation and the second and third PCs 6.75 and 4.20%, respectively. Pea lines were clustered into two major groups on PCs 1 and 2 (Figure 2). The first group included 115 pea lines, among which 91 from the pea core-collection of Bourion et al. (2018). The second group included 145 pea lines, among which 138 were from the pea-Aphanomyces collection. The first three PCs were added to the GWA model as cofactors to take the population structure into account. The Kinship matrix of genetic similarities, also added as cofactor in the GWA model, revealed a moderate relatedness among pea lines of the collection (0.5 < *r*^2^ < 0.75 for most of the lines), as shown in Desgroux et al. (2016) (Supplementary Figure 4).

#### Genome-wide association mapping

**Figure 3:**
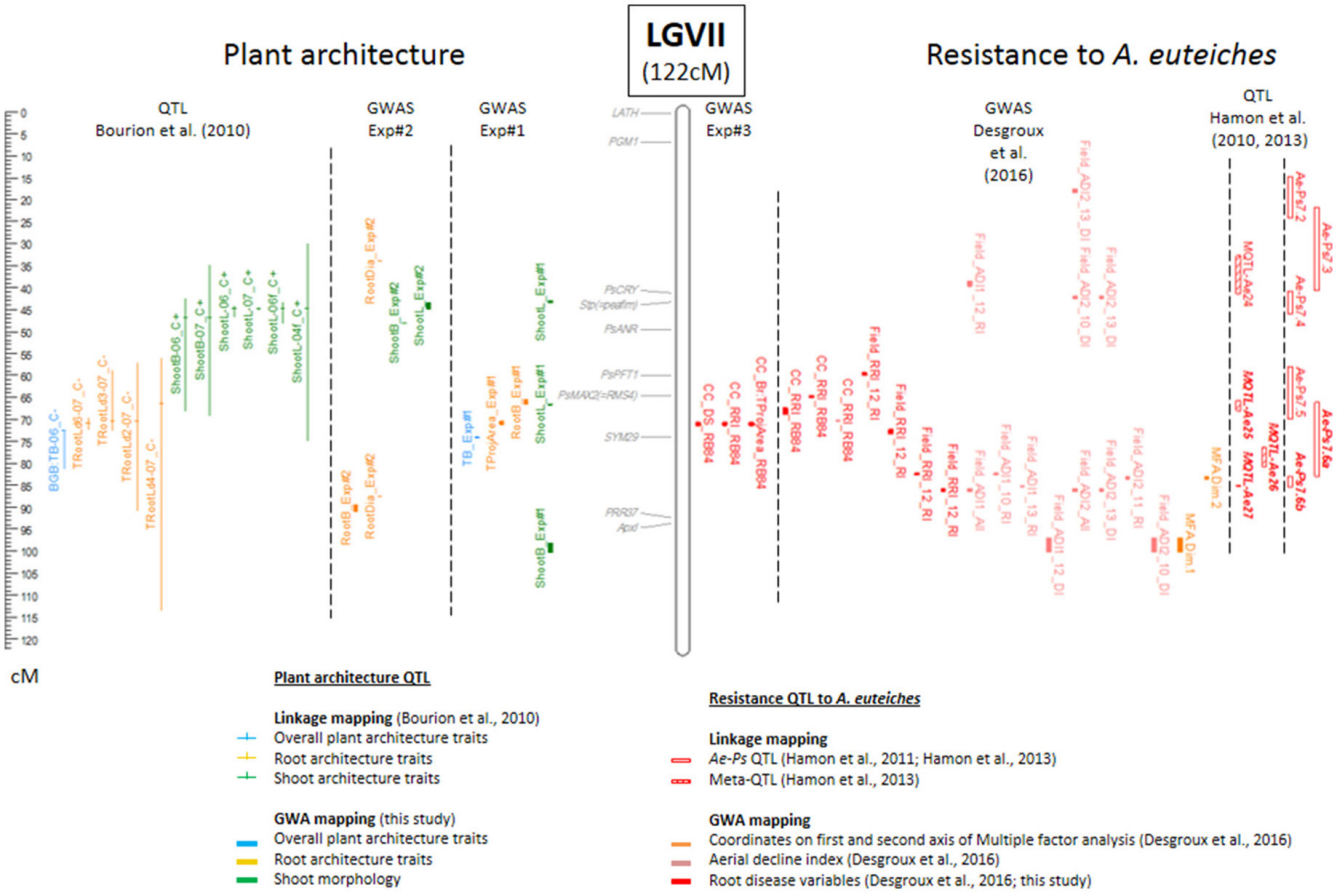
Comparative genetic map of genome-wide association (GWA) and previously detected linkage quantitative trait loci (QTL) for resistance to *A. euteiches* and plant architecture on linkage group VII. The comparative genetic map was constructed from the projection of the 953 markers from Boutet et al. (2016) onto the consensus THMap from Desgroux et al. (2016). Only linkage group (LG) VII is shown (see Supplementary Figure 5 for the other LGs). Its size is indicated in cM Haldane. Shoot architecture-, root architecture-, overall plant architecture—and resistance—associated markers and QTL are indicated in green, orange, blue and red, respectively. To the right of each LG: Confidence intervals (CIs) around significant resistance-associated markers, based on a linkage disequilibrium (LD) value *r*^2^ > 0.2, identified in this study by GWA and name of the trait are indicated (GWAS Exp#3); CIs around significant -resistance-associated markers, identified in controlled conditions by GWA in Desgroux et al. (2016); Projected Meta-QTL (MQTL) and QTL (*Ae-Ps* QTL) described in Hamon et al. (2011) and Hamon et al. (2013), hatched bars represent Meta-QTL, while blank bars represent initial QTL before meta-analysis. The main Aphanomyces root rot resistance QTL and Meta-QTL names are in bold italic. To the left of each LG: Genomic positions of cloned pea genes are indicated in gray; CIs around plant architecture associated markers identified in this study (GWAS Exp#1 and GWAS Exp#2); Projected QTL for root, shoot and plant architecture traits described in Bourion et al. (2010).

A total of 89 SNP markers, distributed over the seven LGs, were significantly associated with phenotypic variation observed in the collection for the plant architecture and disease variables scored in this study (Table 2, Supplementary Table 2, Figure 3, Supplementary Figure 5). Zero to 16 markers were significantly associated with each variable with a *p-value* that ranged from 2.14E-59 to 2.66E-05, depending on the marker and the variable. Over the three experiments, the most significant *p-values* were detected for ShootL and ShootB traits and the less significant for root traits. For each model, the set of markers retained as cofactors explained zero to 73% of the phenotypic variation, depending on the variable and experiment (Table 2). All the 89 significant SNPs corresponded to 85 CIs (Supplementary Table 2). The average size of the CIs defined around the significant SNPs was 0.89 cM, which was lower than that previously observed in Desgroux et al. (2016) (average size = 1.03 cM). Twelve CIs were detected for at least two variables from the same or different experiments.

**Table 2:** Number of markers detected by genome-wide association mapping for plant architecture and Aphanomyces root rot resistance variables.

**Experiment (a)**	**Variable (b)**	**Number of markers (c)**	**Range of *p-value* (d)**	**Range of allelic effect (e)**	**% of phenotypic variance explained by**			**Unexplained variance (i) (%)**
					**PCA (f)**	**Markers (g)**	**Kinship (h)**	
Exp#1	TProjArea	8	2.74E-11–1.68E-05	0.74–1.94	29	35	0	36
	RootDia	0	–	–	35	0	30	35
	TRootL	1	3.29E-06	27.7	18	11	37	34
	NLatRoot	0	–	–	21	0	56	23
	LatRootL	2	5.63E-12–2.21E-06	0.37–0.70	1	26	22	51
	RootB	6	2.12E-08–1.97E-06	2.46–5.68	45	23	4	28
	ShootB	16	5.70E-19–4.05E-06	2.29–8.83	4	73	0	23
	ShootL	10	1.27E-33–2.01E-05	0.46–2.66	48	40	7%	5
	TB	2	4.79E-06–1.08E-05	6.31–9.34	8	20	37	35
	RootB:TB	1	2.23E-07	0.03	68	4	14	14
Exp#2	TProjArea	0	–	–	3	0	76	21
	RootDia	14	5.71E-19–9.22E-06	0.009–0.029	47	36	6	11
	TRootL	3	1.19E-07–2.25E-05	28.89–33.82	4	22	61	13
	NLatRoot	1	4.96E-06	2.81	8	10	70	12
	LatRootL	1	2.66E-05	0.46	3	5	50	42
	RootB	4	6.25E-09–1.97E-05	2.54–6.74	10	24	34	32
	ShootB	12	2.37E-44–1.50E-05	5.42–29.59	3	72	0	25
	ShootL	5	2.14E-59–1.54E-05	0.94–7.96	53	38	7	2
	TB	7	9.04E-18–1.88E-05	7.33–28.19	2	60	10	28
	RootB:TB	2	1.60E-07–1.35E-05	0.01–0.02	23	21	32	24
Exp#3	CC_RRI_RB84	1	2.78E-16	0.33	52	14	17	18
	CC_DS_RB84	5	3.63E-22–2.21E-05	0.12–0.35	51	23	4	22
	CC_Br:TProjArea_RB84	7	6.62E-11–1.57E-05	0.01–0.02	38	28	3	31

(a) Exp#1, Plants were grown in a greenhouse for 8 days before assessment; Exp#2, Plants were grown in a climate controlled chamber for 14 days before assessment; Exp#3, Plants were grown in climate controlled chamber for 14 days and inoculated on the 7th day with a reference strain of A. euteiches (RB84); (b) TProjArea, total root projected area; RootDia, average root diameter; TRootL, total root length; NLatRoot, number of lateral roots; LatRootL, average length of lateral roots; RootB, root biomass; ShootB, shoot biomass; ShootL, shoot length; TB, total biomass; RootB: TB, root to total biomass ratio; CC, controlled conditions; RRI, Root rot index; DS, disease severity score; Br: TProjArea, percentage of brown projected area; (c) Number of markers used as cofactors at the optimal step of the multi-locus mixed model (MLMM) analysis; (d) Range of p-values of the significant markers, significance threshold is p < 2.6E-05 as described in Materials and Methods section; (e) Range of allelic effects at the significant markers, in absolute values; Percentage of phenotypic variance explained by: (f) the principal component analysis (PCA) matrix of the pea collection structure, (g) all significant markers together, (h) the Kinship relatedness matrix among lines of the collection, (i) the unexplained variance qualified as “missing heritability.”

A total of 75 CIs were associated with the variation of one to several of the 10 plant architecture variables scored either in Exp#1 or in Exp#2. Among them, 11 consistent CIs were detected for at least two root, shoot or overall plant architecture variables (Supplementary Table 2, Supplementary Figure 5). Out of the 75 CIs, 32 were specifically identified for RSA traits (LatRootL, NLatRoot, RootB, RootDia, TRootL, TProjArea) (Supplementary Table 2). One of them was consistently detected for three root variables. The associated SNP marker, PsCam007407, on LGIII (31.2 cM), was significantly detected for all the three variables (*p-values* ranging from 3.29E-06 to 5.63E-12, depending on the variable). Thirty other CIs were specifically detected for shoot traits, among which three CIs, on LGIII (27.3 cM near *PsELF3*), LGV (18.6 cM), and LGVII (44 cM), were consistently detected from two or more variables in Exp#1 and/or Exp#2. Four additional CIs were specifically detected from overall plant architecture variables. Six other CIs were associated with both root and shoot or overall plant architecture traits: on LGI (55.1 and 65 cM), LGIII (110 cM near *PsTFL1b*; 132 cM near *PsLE*), LGIV (60.8 cM), and LGV (31.6 cM near *PsTFL1a*). Three other CIs on LGII, LGIII, and LGVI were associated with both shoot and overall architecture traits. The most consistent and significant CI was located in the *PsLE* region on LGIII in which two markers were associated with eight variables scored in Exp#1 and/or Exp#2.

Among the 75 CIs associated with plant architecture variables, 46 overlapped with QTL previously detected for plant architecture by linkage analysis (LA-QTL) in Bourion et al. (2010) (Supplementary Table 2, Figure 3, Supplementary Figure 5). Twenty of the 38 CIs detected in our study for root variables overlapped with LA-QTL previously detected for at least root variables, and five others with only shoot or overall LA-QTL. Among the 20 overlapping CIs, 12 involved consistent root LA-QTL, on LGI (81.2 cM), on LGIII close to *PsTFL1b* and to *PsLE*, on LGIV (0.7; 2 and 16.2 cM), on LGV (close to *PsTFL1a* and 41.1 cM*)*, and on LGVII (66.2; 71.3; 87.9 and 91.2 cM). Similarly, 18 of the CIs detected for shoot and overall variables overlapped with LA-QTL previously detected for at least shoot and/or overall variables, and eight others overlapped with only root LA-QTL.

Eleven CIs were identified for resistance to *A. euteiches* (RRI, DS, Br:TProjArea variables) from Exp#3 (Supplementary Table 2). The *Ps115429* SNP marker on the CI in LGVII (71.3 cM) was highly significantly detected for all the three disease variables (*p values* ranged from 6.62E-11 to 3.63E-22, depending on the variable). This CI overlapped with *Ae-Ps7.6*, the most consistent QTL previously detected for resistance to *A. euteiches* (Hamon et al., 2013), and mapped precisely to a CI (LD block VII.7) associated with resistance to *A. euteiches* previously identified in Desgroux et al. (2016) from controlled condition data with the same RB84 *A. euteiches* strain as used in this study. It is the same region as four other CIs also detected by Desgroux et al. (2016) in controlled conditions (LD blocks VII.8 and VII.10) or in the field (LD blocks VII.6 and VII.11). Six other significant SNPs co-localized with other consistent (*Ae-Ps5.1, Ae-Ps4*.5) or less consistent (*Ae-Ps1.1, Ae-Ps2.1, Ae-Ps3.2, Ae-Ps6.1*) genomic regions previously identified by Hamon et al. (2013). However, four significant SNPs also localized to regions that were not previously identified by linkage analysis (Supplementary Table 2, Figure 3, Supplementary Figure 5).

#### Comparative mapping of plant architecture and aphanomyces root rot resistance loci

Among the 75 and 11 CIs identified in this study for plant architecture and Aphanomyces root rot resistance traits, respectively, one CI (LGVII, 71.3 cM) was commonly detected for both trait types. At this CI, the SNP marker *Ps115429* was associated with variation in resistance for all the three variables studied and with variation of a RSA trait (TProjArea-Exp#1). At this marker, opposing allelic effects for RSA and disease variables were observed, i.e., alleles contributing to a higher root projected area contributed to a smaller disease score (increased resistance; Supplementary Table 2). This CI overlapped with consistent LA-QTL previously detected for total root length variation at different young stages (Bourion et al., 2010) and resistance to *A. euteiches* (Hamon et al., 2013; Desgroux et al., 2016).

Compared with previous GWAS results from Desgroux et al. (2016), seven additional plant architecture CIs detected in this study overlapped with CIs previously associated with field aerial or root resistance to *A. euteiches*. This included six CIs located in the *Ae-Ps2.2, Ae-Ps3.1, Ae-Ps5.2*, and *Ae-Ps6.1* QTL regions. Three of the seven CIs were associated with RSA variables and the other three were associated with shoot and/or overall plant architecture variables (Supplementary Table 2).

When compared with previous QTL linkage analysis results, five other CIs detected from disease severity measurements on roots (Br:TProjArea or DS) overlapped with LA-QTL previously associated with variation of TRootL, NLatRoot, and/or RootB on LGII (52.2 cM), LGIII (89.4 cM, *Ae-Ps3.2* and 132 cM), and LGV (29.8 cM, *Ae-Ps5.1* and 37.8 cM) (Bourion et al. (2010). Another CI for Br:TProjArea overlapped with a consistent LA-QTL for shoot variables on LGII (472.4 cM). Fourty-eight other CIs detected for plant architecture in this study overlapped with LA-QTL previously reported for resistance to *A. euteiches* in Hamon et al. (2013).

## Discussion

In this study, the diversity of loci involved in plant architecture, especially of the root system, in pea at young stages was explored and compared with loci for resistance to a major root disease, Aphanomyces root rot. This study was based on a GWA approach, using a collection of 266 pea lines established from previous collections including contrasted lines for plant architecture traits and Aphanomyces root rot resistance. It used precise phenotyping methods, based on image analysis, to characterize plant architecture and resistance in roots. Innovative results were obtained about precise comparative mapping of genetic loci and alleles associated with plant architecture and resistance to *A. euteiches*, opening prospects for mining root architecture loci in breeding to limit Aphanomyces root rot severity in peas.

### High genetic and QTL diversity for plant architecture

In the two experiments conducted in this study in different pathogen-free controlled conditions, we confirmed the high diversity in aerial plant architecture in *P. sativum*. However, our findings also provided new insight into pea root system diversity. The first study on root biomass and RSA diversity in pea reported a high diversity based on observation, 14 days post sowing, of a collection of 330 accessions (McPhee, 2005). Bourion et al. (2010) identified genetic diversity in shoot and RSA traits among seven pea lines from the four-leaf stage to the beginning of seed filling, and also investigated the genetic determinism of these traits through analysis of a RIL pea population phenotyped from two until 27 days post-germination. In our study, we confirmed that the genetic diversity of pea plant architecture was observable at the early stages of 8 and 14 days after sowing and we found an even wider range of values for some traits (ShootB, RootB, TRootL) than observed by McPhee (2005). We also found that the 8- and 14-day architectures were highly correlated (*r* > 0.38) in the two different experiments. Thus, we confirmed that plant architecture is set up very early in the plant development process.

Our GWA study highlighted the high number of genetic factors that control shoot and root pea architecture traits, in agreement with Bourion et al. (2010). We confirmed most of the previous QTL and identified them with higher accuracy, since 11 of the LA-QTL previously detected in Bourion et al. (2010) overlapped with 46 CIs significantly detected in this study. In addition, due to the large variability screened in the 266-pea-line collection, our study allowed new plant architecture loci to be identified. Indeed, 29 CIs detected in this study did not overlap with previous LA-QTL. These results provide closely-linked SNP markers or new loci controlling plant shoot and root architecture for marker-assisted-selection.

### Several genes determining plant shoot architecture or flowering also have putative pleiotropic effects on root architecture

Using annotation data from Tayeh et al. (2015), we could identify several putative genes from the SNP-anchored sequences in the main LD blocks associated with plant architecture variation in this study. As such, three SNP markers associated with the most consistent and significant shoot architecture variation were designed either in the sequence or in the same LD block as three cloned pea genes, i.e., *PsLE* which encodes gibberellin 3b-hydroxylase and controls inter-node length, and *PsTFL1a* and *PsTFL1b* both involved in flowering regulation. These three SNPs were also detected for root biomass variation, with allelic effects of the same sign on both shoots and roots. These SNPs were located in CIs which overlapped with LA-QTL previously detected for root variables by Bourion et al. (2010). These results confirm the pleiotropic effect of the major developmental gene *PsLE* previously observed in roots (Weeden and Moffet, 2002; Bourion et al., 2010). They also suggest a pleiotropic effect for genes involved in flowering regulation, even at early developmental stages. *PsTFL1a* ( = *DET*) is known to induce a reduction in the flowering period and thus a determinate growth habit (Foucher et al., 2003). Determinate dry pea cultivars have been preferred to indeterminate ones because their time of growth coincides better with the soil–moisture–availability and their lower lodging allows easier mechanical seed harvesting (Duc et al., 2015). Selection of determinacy in shoot growth or accelerated flowering traits could also have an impact on the growth potential of roots. This effect could be either indirect, as an adaptation to a decrease in carbon from the shoot or direct, through the common control of aerial and root growth. As an example, *PsTFL1a* is expressed in the shoot apex only after the transition to flowering and is expressed in roots regardless of the developmental stage. *PsTFL1b* expression was never found in flowers but it is expressed in roots and the shoot apex during both vegetative and reproductive stages (Foucher et al., 2003). Thus, the consistency of the significant CIs identified here, at the *PsTFL1* genes, suggests that regions controlling flowering and shoot length were also involved, at least indirectly, in the regulation of root traits.

### Other genes are putatively involved in either root or shoot architecture

Several other CI regions were consistently found to be specifically associated with root architecture, within this study or with the previous LA-QTL study (Bourion et al., 2010). One was associated with a SNP marker, PSCam029391 (LGIV, 2.0 cM), located in a gene specifically expressed in roots or nodules and encoding a putative Rho GDP dissociation inhibitor (Alves-Carvalho et al., 2015). Rho GDP dissociation inhibitors play an important role in regulating the activity of Rho GTPases, which have been found to be localized to root hair tips where they control polar root growth in *Arabidopsis thaliana* (Bischoff et al., 2000; Molendijk et al., 2001). Other consistent CIs related to root variation were found to be associated with SNP markers in genes encoding for phosphatidic acid (PA) hydrolase; among them, the CI detected for three root variables in this study (LGIII, PsCam007407). PA is an essential phospholipid involved in membrane biosynthesis and has a messenging role during plant stress, metabolism, and development (Testerink and Munnik, 2011). Other interesting loci are those involved in RootDia variation on LGII found to be associated with SNP markers in genes encoding for either polygalacturonase (PG) or PG-inhibiting protein (PGIP). PG is one of the most important enzymes involved in plant cell wall degradation. PGIPs are extracellular leucine-rich repeat proteins that recognize and inhibit fungal PGs. The PG–PGIP interaction favors the accumulation of elicitor-active oligogalacturonides and causes the activation of defense responses (Federici et al., 2006).

Consistent genomic regions on LGIII (27.3 cM), LGV (18.6 cM), and LGVII (43.6 cM) were also specifically associated with shoot variation. The first two were newly identified in contrast to the LA-QTL previously identified in Bourion et al. (2010). The SNP marker (PsCam038460) on LGIII was found to correspond to a gene mainly expressed in the stem. The LGV region included two SNP markers, PsCam001152 and PsCam056107, both in genes highly specifically expressed in shoot and leaves, and encoding a chloroplast RNA binding protein and a Photosystem II protein, respectively (Alves-Carvalho et al., 2015). In the third region, identified on LGVII, the associated SNP marker (PsCam044214) was in a gene encoding a putative chloroplast protein and thus highly expressed in shoot but not in roots. Thus, our findings highlight that genes involved in the photosynthesis process have little direct effects on root traits.

### Root architecture traits correlated with resistance to *A. euteiches*

In our study, we confirmed the large diversity of plant responses to *A. euteiches* as well as the high levels of resistance previously reported in several pea lines (Desgroux et al., 2016). As *A. euteiches* infection occurs at the seedling stage, putative RSA and resistance relationships should be established from the first plant development stages. Indeed, most of the RSA traits were negatively correlated with Aphanomyces root rot susceptibility traits in our study. Correlations indicated that the resistance was related to increased root architecture features (root length, number of lateral roots, root biomass) before infection. Among the most resistant pea lines, *AeD99OSW-58-10-5* and *AeD99OSW-50-2-5* in particular showed high root system bushiness (longer root system and higher number of lateral roots). However, some highly resistant pea lines had moderate root system bushiness, such as *AeD99OSW-45-8-7*. In similar studies on the bean-Fusarium root rot pathosystem, a larger number of lateral roots was correlated with an increased level of resistance (Román-Avilés et al., 2004). Kraft and Boge (2001) also demonstrated on 12 pea lines that large-rooted (large total root length) pea lines were less susceptible to *Fusarium solani* in infested fields. A wide, long and branched root system was shown to be related to an increased tolerance to vine collapse in melon (Dias et al., 2004). In our study, the average root diameter tended to be higher in pea lines that were susceptible to *A. euteiches*, in correlation with a lower root length. However, in other studies, such as in the bean-*Fusarium solani* pathosystem (Snapp et al., 2003), a larger root diameter was reported to be associated with a higher level of resistance.

Interestingly, in aerial plant/pathogen pathosystems, higher shoot density was more often correlated with higher susceptibility (Calonnec et al., 2013). The processes involved in pathogen development and disease epidemics can be different for the above- and below-ground parts of the plant. For aerial organs, dense architecture could create moisture and a microclimate favorable to disease development (Richard et al., 2013). Whereas, for roots, increased density could enhance the plant's ability to draw resources from the soil or limit pathogen root colonization as previously suggested (Djébali et al., 2009). Indeed, it would probably take more time for a pathogen to colonize and develop symptoms on larger root systems than on smaller ones. In addition, pea lines with a larger root projected area before infection may also retain a higher amount of healthy root segments to continue to capture water and nutrients and to produce new root segments, under disease pressure.

### Common genetic loci associated with root system architecture and resistance to *A. euteiches*

In this study, comparative GWA analysis accurately identified a total of eight CIs associated with both plant architecture and resistance to *A. euteiches*. When compared to previous QTL linkage analysis conducted for these traits (Bourion et al., 2010; Hamon et al., 2013), many more overlapping intervals were identified between the resistance and plant architecture traits. The high resolution QTL detection and low-size CIs defined in GWA approaches resulted in more reliable GWA-QTL co-localizations than the LA-QTL coincident regions detected from Bourion et al. (2010) and Hamon et al. (2013). Among the co-localizing plant architecture and resistance loci, one major locus was identified from assays conducted in controlled conditions in this study. The seven other loci, associated with plant architecture in this study, were previously detected by GWAS for field aerial or root resistance to *A. euteiches* (Desgroux et al., 2016). Some were located in genomic regions containing genes controlling plant development (*PsELF3* on LGIII), which may suggest possible pleiotropic effects of plant architecture genes on resistance or tolerance traits, as reported in Poland et al. (2009). In other legumes, co-localization between root rot resistance and RSA loci have been reported. In linkage analysis in common bean, Hagerty et al. (2015) found overlapping QTLs controlling Aphanomyces root rot resistance and taproot diameter on chromosome Pv02. The authors observed an association between increased taproot and resistance. In black bean, a co-localization was identified on chromosome Pv05 between QTL controlling deep root weight, total plant biomass and resistance to Fusarium root rot detected by linkage analysis (Nakedde et al., 2016).

In our study, the major locus associated with both resistance (RRI, DS and Br:TProjArea) and RSA (TProjArea) traits was revealed by one highly significant SNP marker, i.e., *Ps115429*. It was located in the same genomic region as the major QTL *Ae-Ps7.6* previously detected by linkage (Hamon et al., 2013) and GWA mapping (Desgroux et al., 2016), the effect of which was validated in Near-Isogenic Lines (Lavaud et al., 2015). At this marker, the resistance-enhancing allele was associated with an increased total root projected area. No previous study identified such a precise co-localization of root disease resistance and RSA traits. In this study, by using SNP markers from Boutet et al. (2016) we could pinpoint the genetic position of this Aphanomyces resistance-associated locus, which makes it interesting for marker-assisted-selection. The SNP sequence of *Ps115429* mapped to an intron in the MTR_4g074875 gene coding for a MAP kinase on the *M. truncatula* genome. MAP-kinases are known to be involved in plant immunity triggered by pathogen effectors (Rasmussen et al., 2012). In wheat, a gene coding for a MAP-kinase was involved in tolerance to several abiotic stresses as well as root growth (Hao et al., 2015). Our findings suggest that a MAP-kinase gene could have a pleiotropic effect on both root growth and resistance to *A. euteiches*, even though it is possible that closely-linked genes may underly the major locus associated with both traits. Selecting pea lines for this major locus could thus improve both disease resistance and root system bushiness, conferring an advantage to the plant through improved water and nutrient retention.

## Conclusion

This study provides new results for a better understanding of plant architecture genetic determinism and genetic interdependency of root disease resistance and RSA inheritance. Pea lines with good levels of resistance to *A. euteiches* and a large root system (larger number of roots and longer roots) were identified and could be useful for breeders to improve resistance to *A. euteiches* in pea varieties. A SNP marker, detected for both improved resistance to *A. euteiches* and high projected root area will be relevant for use in the marker-assisted-selection of resistant varieties. Other genetic loci associated with both plant architecture and resistance would be of interest for breeding architectural pea types limiting disease development. Further studies will be useful to validate pleiotropy effect of candidate genes underlying loci associated with plant root architecture and disease resistance.

## Author contributions

AD managed and participated in phenotypic data acquisition, performed all the phenotypic and GWA analyses and drafted the manuscript. VNB, VA, GLR, HdL, and HM contributed to implement controlled condition assays, evaluated plant architecture and disease resistance traits and analyzed root images. GA and GB coordinated the SNP genotyping of the collection, performed its analysis and contributed to genetic analysis. GB and AB supervised the KASPar SNP design program. JB designed and coordinated the Infinium® BeadChip genotyping program of the pea-collection. AB, JB, and GD provided scientific expertise on the conception of the study. MM-D co-supervised the conception of the study and the drafting of the manuscript. M-LP-N and VB coordinated the overall study and the manuscript draft. All authors read and approved the final manuscript.

### Conflict of interest statement

The authors declare that the research was conducted in the absence of any commercial or financial relationships that could be construed as a potential conflict of interest.

## Supplementary material

Supplementary Figure 1 Frequency distribution of least square means obtained for root, shoot and overall plant architecture variables, and for Aphanomyces root rot resistance in the 266-pea-line collection. Least square means were obtained from analysis of variance for six root system architecture variables (total projected area, average root diameter, total root length, number of lateral roots, average length of lateral roots, root biomass, coded as TProjArea, RootDia, TRootL, NLatRoot, LatRootL, RootB, respectively), two shoot architecture variables (shoot biomass and shoot length, coded as ShootB and ShootL, respectively), two overall plant architecture variables (total biomass and root to total biomass ratio, coded as TB and RootB:TB, respectively) and three Aphanomyces root rot resistance variables (disease severity, root rot index and percentage of brown projected area, coded DS, RRI and Br:TProjArea, respectively). Architectural traits were assessed on 8 day-old plants in a greenhouse (Exp#1) and 14 day-old plants in a climate controlled chamber (Exp#2). Aphanomyces resistance traits were assessed on 14 day-old plants inoculated at the 7th day with a reference strain of *A. euteiches* (RB84) in a climate controlled chamber (Exp#3). n: total number of pea lines assessed; m: mean ± standard deviation of the 266-pea-line collection; h^2^: mean-based heritability.

Supplementary Figure 2 Regression line between disease severity score and percentage of brown projected area measured in Exp#3. LSMeans of disease severity (DS) score was plotted against LSMeans of percentage of brown projected area (Br:TProjArea). Function of the regression line (y = 0.25804x + 0.06497) was calculated through the linear model Br:TProjArea DS. The regression coefficient *r*^2^ translates the relevance of the model.

Supplementary Figure 3 Linkage disequilibrium (LD) decay in the 266-pea-line collection. Colored curves represent the estimated LD decay for each linkage group (LG). Dashed vertical lines represent the LD threshold (maximum *r*^2^/2) and arrows the LD decay rate, as the estimated genetic distance (cM) to reach this LD threshold on each LG.

Supplementary Figure 4 Clustered heatmap of the Kinship matrix of the 266-pea-line collection.Kinship matrix from the GAPIT R package (Lipka et al., 2012) based on 2,937 SNP markers. Clustering is based on the UPGMA method. The color gradient represents the degree of relationship between two lines shown. Kinship matrix from the GAPIT R package (Lipka et al., 2012) based on 2,937 SNP markers. Clustering is based on the UPGMA method. The color gradient represents the degree of relationship between two lines shown.

Supplementary Figure 5 Comparative genetic map of genome-wide association (GWA) and previously detected linkage quantitative trait loci (QTL) for resistance to *A. euteiches* and plant architecture on LGI to VI. The comparative genetic map was constructed from the projection of the 953 markers from Boutet et al. (2016) onto the consensus THMap from Desgroux et al. (2016). Linkage groups (LG) I to VI are shown (see Figure 3 for LGVII). LG size is indicated in cM Haldane. Shoot architecture-, root architecture-, overall plant architecture- and resistance- associated markers and QTL are indicated in green, orange, blue and red, respectively. To the right of each LG: Confidence intervals (CIs) around significant resistance-associated markers, based on linkage disequilibrium (LD) value *r*^2^ > 0.2, identified in this study by GWA and name of the trait are indicated (GWAS Exp#3); CIs around significant resistance-associated markers, identified in controlled conditions by GWA in Desgroux et al. (2016); Projected Meta-QTL (MQTL) and QTL (*Ae-Ps* QTL) described in Hamon et al. (2011) and Hamon et al. (2013), hatched bars represent Meta-QTL, while blank bars represent initial QTL before meta-analysis. The main Aphanomyces root rot resistance QTL and Meta-QTL names are in bold italic. To the left of each LG: Genomic positions of cloned pea genes are indicated in gray; CIs around plant architecture associated markers identified in this study (GWAS Exp#1 and GWAS Exp#2); Projected QTL for root, shoot and plant architecture traits described in Bourion et al. (2010).

Supplementary Table 1 Statistical analysis results of plant architecture and Aphanomyces root rot resistance data in the 266-pea-line collection.

Supplementary Table 2 Genetic markers associated with plant architecture and Aphanomyces root rot resistance variables detected by genome-wide association mapping.

## Abbreviations

Br:TProjArea: percentage of root system with browning symptoms
CIs: confidence intervals
DS: disease severity
GWA: genome wide association
LA: linkage analysis
LatRootL: average lateral root length
LD: linkage disequilibrium
LG: linkage group
LM: linear model
MAF: minor allele frequency
mBonf: multiple-Bonferroni
MLMM: multi-locus mixed model
NLatRoot: number of first lateral roots
PCA: Principal Component Analysis
RIL: recombinant inbred line
RootB: root biomass
RootB:TB: root to total biomass ratio
RootDia: root diameter
RRI: root rot index
RSA: root system architecture
ShootB: shoot biomass
ShootL: plant height
SNP: single nucleotide polymorphism
TB: total biomass
TProjArea: total root projected area
TRootL: total root length
QTL: quantitative trait loci.

## References

[B1] Alves-Carvalho S. Aubert G. Carrère S. Cruaud C. Brochot A.-L. Jacquin F.. (2015). Full-length *de novo* assembly of RNA-seq data in pea (*Pisum sativum* L.) provides a gene expression atlas and gives insights into root nodulation in this species. Plant J. 84, 1–19. 10.1111/tpj.12967 26296678

[B2] Benjamini Y. Hochberg Y. (1995). Controlling the false discovery rate: a practical and powerful approach to multiple testing. J. R. Statist. Soc. B 57, 289–300.

[B3] Bischoff F. Vahlkamp L. Molendijk A. Palme K. (2000). Localization of AtROP4 and AtROP6 and interaction with the guanine nucleotide dissociation inhibitor AtRhoGDI1 from Arabidopsis. Plant Mol. Biol. 42, 515–530. 10.1023/A:1006341210147 10798620

[B4] Bonhomme M. André O. Badis Y. Ronfort J. Burgarella C. Chantret N.. (2014). High-density genome-wide association mapping implicates an F-box encoding gene in *Medicago truncatula* resistance to *Aphanomyces euteiches*. New Phytologist 201, 1328–1342. 10.1111/nph.12611 24283472

[B5] Bourion V. Heulin-Gotty K. Aubert V. Tisseyre P. Chabert-Martinello M. Pervent M. (2018). Co-inoculation of a pea core-collection with diverse rhizobial strains shows competitiveness for nodulation efficiency of nitrogen fixation are distinct traits in the interaction. Front. Plant Sci. 8:2249 10.3389/fpls.2017.02249 PMC5767787 29367857

[B6] Bourion V. Rizvi S. M. H. Fournier S. de Larambergue H. Galmiche F. Marget P.. (2010). Genetic dissection of nitrogen nutrition in pea through a QTL approach of root, nodule, and shoot variability. Theor. Appl. Genet. 121, 71–86. 10.1007/s00122-010-1292-y 20180092

[B7] Boutet G. Alves Carvalho S. Falque M. Peterlongo P. Lhuillier E. Bouchez O.. (2016). SNP discovery and genetic mapping using genotyping by sequencing of whole genome genomic DNA from a pea RIL population. BMC Genomics 17:121. 10.1186/s12864-016-2447-2 26892170 PMC4758021

[B8] Calonnec A. Burie J.-B. Langlais M. Guyader S. Saint-Jean S. Sache I. (2013). Impacts of plant growth and architecture on pathogen processes and their consequences for epidemic behaviour. Eur. J. Plant Pathol. 135, 479–497. 10.1007/s10658-012-0111-5

[B9] Chang C. C. Chow C. C. Tellier L. C. Vattikuti S. Purcell S. M. Lee J. J. (2015). Second-generation PLINK: rising to the challenge of larger and richer datasets. Gigascience 4:7. 10.1186/s13742-015-0047-8 25722852 PMC4342193

[B10] Cichy K. A. Snapp S. S. Kirk W. W. (2007). Fusarium root rot incidence and root system architecture in grafted common bean lines. Plant Soil 300, 233–244. 10.1007/s11104-007-9408-0

[B11] Desgroux A. L'Anthoëne V. Roux-Duparque M. Rivière J.-P. Aubert G. Tayeh N.. (2016). Genome-wide association mapping of partial resistance to *Aphanomyces euteiches* in pea. BMC Genomics 17:124. 10.1186/s12864-016-2429-4 26897486 PMC4761183

[B12] Dias R. d. C. S. Picó B. Espinos A. Nuez F. (2004). Resistance to melon vine decline derived from *Cucumis melo* ssp. agrestis: genetic analysis of root structure and root response. Plant Breed. 123, 66–72. 10.1046/j.1439-0523.2003.00944.x

[B13] Djébali N. Aribi S. Taamalli W. Arraouadi S. Aouani M. E. Badri M. (2013). Natural variation of *Medicago truncatula* resistance to *Aphanomyces euteiches*. Eur. J. Plant Pathol. 135, 831–843. 10.1007/s10658-012-0127-x

[B14] Djébali N. Jauneau A. Ameline-Torregrosa C. Chardon F. Jaulneau V. Mathe C.. (2009). Partial resistance of *Medicago truncatula* to *Aphanomyces euteiches* is associated with protection of the root stele and is controlled by a major QTL rich in proteasome-related genes. Mol. Plant Microbe Int. 22, 1043–1055. 10.1094/MPMI-22-9-1043 19656040

[B15] Downie H. F. Adu M. O. Schmidt S. Otten W. Dupuy L. X. White P. J.. (2015). Challenges and opportunities for quantifying roots and rhizosphere interactions through imaging and image analysis. Plant Cell Environ. 38, 1213–1232. 10.1111/pce.12448 25211059

[B16] Duc G. Agrama H. Bao S. Y. Berger J. Bourion V. De Ron A. M. (2015). Breeding annual grain legumes for sustainable agriculture: new methods to approach complex traits and target new cultivar ideotypes. Crit. Rev. Plant Sci. 34, 381–411. 10.1080/07352689.2014.898469

[B17] Federici L. Di Matteo A. Fernandez-Recio J. Tsernoglou D. Cervone F. (2006). Polygalacturonase inhibiting proteins: players in plant innate immunity? Trends Plant Sci. 11, 65–70. 10.1016/j.tplants.2005.12.005 16406303

[B18] Foucher F. Morin J. Courtiade J. Cadioux S. Ellis N. Banfield M. J.. (2003). DETERMINATE and LATE FLOWERING are two TERMINAL FLOWER1/CENTRORADIALIS homologs that control two distinct phases of flowering initiation and development in pea. Plant Cell 15, 2742–2754. 10.1105/tpc.015701 14563931 PMC280576

[B19] Gaulin E. Jacquet C. Bottin A. Dumas B. (2007). Root rot disease of legumes caused by *Aphanomyces euteiches*. Mol. Plant Pathol. 8, 539–548. 10.1111/j.1364-3703.2007.00413.x 20507520

[B20] Gupta P. K. Kulwal P. L. Jaiswal V. (2014). Association mapping in crop plants: opportunities and challenges. Adv. Genet. 85, 109–147. 10.1016/B978-0-12-800271-1.00002-0 24880734

[B21] Hagerty C. H. Cuesta-Marcos A. Cregan P. B. Song Q. McClean P. Noffsinger S. (2015). Mapping *Fusarium solani* and *Aphanomyces euteiches* root rot resistance and root architecture Quantitative Trait Loci in common bean. Crop Sci. 55, 1969–1977. 10.2135/cropsci2014.11.0805

[B22] Hamon C. Baranger A. Coyne C. J. McGee R. J. Le Goff I. L'Anthoëne V.. (2011). New consistent QTL in pea associated with partial resistance to *Aphanomyces euteiches* in multiple French and American environments. Theor. Appl. Genet. 123, 261–281. 10.1007/s00122-011-1582-z 21479935

[B23] Hamon C. Coyne C. J. McGee R. J. Lesné A. Esnault R. Mangin P.. (2013). QTL meta-analysis provides a comprehensive view of loci controlling partial resistance to *Aphanomyces euteiches* in four sources of resistance in pea. BMC Plant Biol. 13:45. 10.1186/1471-2229-13-45 23497245 PMC3680057

[B24] Hao L. Wen Y. Zhao Y. Lu W. Xiao K. (2015). Wheat mitogen-activated protein kinase gene TaMPK4 improves plant tolerance to multiple stresses through modifying root growth, ROS metabolism, and nutrient acquisitions. Plant Cell Rep. 34, 2081–2097. 10.1007/s00299-015-1853-2 26275989

[B25] Hodge A. Berta G. Doussan C. Merchan F. Crespi M. (2009). Plant root growth, architecture and function. Plant Soil 321, 153–187. 10.1007/s11104-009-9929-9

[B26] Kraft J. M. Boge W. (2001). Root characteristics in pea in relation to compaction and Fusarium root rot. Plant Dis. 85, 936–940. 10.1094/PDIS.2001.85.9.936 30823105

[B27] Laffont C. Rey T. Andre O. Novero M. Kazmierczak T. Debelle F.. (2015). The CRE1 cytokinin pathway is differentially recruited depending on *Medicago truncatula* root environments and negatively regulates resistance to a pathogen. PLoS ONE 10:e0116819. 10.1371/journal.pone.0116819 25562779 PMC4285552

[B28] Lavaud C. Lesné A. Piriou C. Le Roy G. Boutet G. Moussart A.. (2015). Validation of QTL for resistance to *Aphanomyces euteiches* in different pea genetic backgrounds using near-isogenic lines. Theor. Appl. Genet. 128, 2273–2288. 10.1007/s00122-015-2583-0 26215183

[B29] Lenth R. V. Hervé M. (2015). lsmeans: Least-Squares Means. R package version 2.15. Available online at: http://CRAN.R-project.org/package=lsmeans

[B30] Lipka A. E. Tian F. Wang Q. Peiffer J. Li M. Bradbury P. J.. (2012). GAPIT: genome association and prediction integrated tool. Bioinformatics 28, 2397–2399. 10.1093/bioinformatics/bts444 22796960

[B31] Malamy J. E. (2005). Intrinsic and environmental response pathways that regulate root system architecture. Plant Cell Environ. 28, 67–77. 10.1111/j.1365-3040.2005.01306.x 16021787

[B32] McDonald M. R. Gossen B. D. Kora C. Parker M. Boland G. (2013). Using crop canopy modification to manage plant diseases. Eur. J. Plant Pathol. 135, 581–593. 10.1007/s10658-012-0133-z

[B33] McPhee K. (2005). Variation for seedling root architecture in the core collection of pea germplasm. Crop Sci. 45, 1758–1763. 10.2135/cropsci2004.0544

[B34] Molendijk A. J. Bischoff F. Rajendrakumar C. S. V. Friml J. Braun M. Gilroy S.. (2001). Arabidopsis thaliana Rop GTPases are localized to tips of root hairs and control polar growth. EMBO J. 20, 2779–2788. 10.1093/emboj/20.11.2779 11387211 PMC125484

[B35] Moussart A. Wicker E. Duparque M. Rouxel F. (2001). Development of an efficient screening test for pea resistance to *Aphanomyces euteiches*, in 4th European Conference On Grain Legumes, ed AEP), 272–273.

[B36] Nakedde T. Ibarra-Perez F. J. Mukankusi C. Waines J. G. Kelly J. D. (2016). Mapping of QTL associated with Fusarium root rot resistance and root architecture traits in black beans. Euphytica 212, 51–63. 10.1007/s10681-016-1755-6

[B37] Ney B. Bancal M. O. Bancal P. Bingham I. J. Foulkes J. Gouache D. (2013). Crop architecture and crop tolerance to fungal diseases and insect herbivory. Mechanisms to limit crop losses. Eur. J. Plant Pathol. 135, 561–580. 10.1007/s10658-012-0125-z

[B38] Pascual L. Albert E. Sauvage C. Duangjit J. Bouchet J.-P. Bitton F.. (2016). Dissecting quantitative trait variation in the resequencing era: complementarity of bi-parental, multi-parental and a*sso*ciation panels. Plant Sci. 242, 120–130. 10.1016/j.plantsci.2015.06.017 26566830

[B39] Pilet-Nayel M. L. Muehlbauer F. J. McGee R. J. Kraft J. M. Baranger A. Coyne C. J. (2005). Consistent quantitative trait loci in pea for partial resistance to *Aphanomyces euteiches* isolates from the United States and France. Phytopathology 95, 1287–1293. 10.1094/PHYTO-95-1287 18943359

[B40] Pilet-Nayel L. Muehlbauer F. J. McGee R. J. Kraft J. M. Baranger A. Coyne C. J. (2002). Quantitative trait loci for partial resistance to Aphanomyces root rot in pea. Theor. Appl. Genet. 106, 28–39. 10.1007/s00122-002-0985-2 12582868

[B41] Poland J. A. Balint-Kurti P. J. Wisser R. J. Pratt R. C. Nelson R. J. (2009). Shades of gray: the world of quantitative disease resistance. Trends Plant Sci. 14, 21–29. 10.1016/j.tplants.2008.10.006 19062327

[B42] Purcell S. Neale B. Todd-Brown K. Thomas L. Ferreira M. A. Bender D.. (2007). PLINK: a tool set for whole-genome association and population-based linkage analyses. Am. J. Hum. Genet. 81, 559–575. 10.1086/519795 17701901 PMC1950838

[B43] Rasmussen M. W. Roux M. Petersen M. Mundy J. (2012). MAP kinase cascades in Arabidopsis innate immunity. Front. Plant Sci. 3:169. 10.3389/fpls.2012.00169 22837762 PMC3402898

[B44] R Core Team (2014). R: A Language and Environment for Statistical Computing. R Foundation for Statistical Computing.

[B45] Revelle W. (2015). psych: Procedures for Psychological, Psychometris and Personality Research. R package version 1.5.1. Available online at: http://CRAN.R-project.org/package=psych

[B46] Richard B. Bussière F. Langrume C. Rouault F. Jumel S. Faivre R. (2013). Effect of pea canopy architecture on microclimate and consequences on ascochyta blight infection under field conditions. Eur. J. Plant Pathol. 135, 509–524. 10.1007/s10658-012-0132-0

[B47] Román-Avilés B. Snapp S. S. Kelly J. D. (2004). Assessing root traits associated with root rot resistance in common bean. Field Crops Res. 86, 147–156. 10.1016/j.fcr.2003.08.001

[B48] Schwender H. Fritsch A. (2013). scrime: Analysis of High-Dimensional Categorical Data Such as SNP data. R package version 1.3.3. Available online at: http://CRAN.R-project.org/package=scrime

[B49] Segura V. Vilhjalmsson B. J. Platt A. Korte A. Seren U. Long Q.. (2012). An efficient multi-locus mixed-model approach for genome-wide association studies in structured populations. Nat. Genet. 44, 825–830. 10.1038/ng.2314 22706313 PMC3386481

[B50] Snapp S. Kirk W. Román-Avilés B. Kelly J. (2003). Root traits play a role in integrated management of Fusarium root rot in snap beans. HortScience 38, 187–191.

[B51] Sosnowski O. Charcosset A. Joets J. (2012). BioMercator V3: an upgrade of genetic map compilation and quantitative trait loci meta-analysis algorithms. Bioinformatics 28, 2082–2083. 10.1093/bioinformatics/bts313 22661647 PMC3400960

[B52] Tayeh N. Aluome C. Falque M. Jacquin F. Klein A. Chauveau A.. (2015). Development of two major resources for pea genomics: the GenoPea 13.2K SNP Array and a high-density, high-resolution consensus genetic map. Plant J. 84, 1257–1273. 10.1111/tpj.13070 26590015

[B53] Testerink C. Munnik T. (2011). Molecular, cellular, and physiological responses to phosphatidic acid formation in plants. J. Exp. Bot. 62, 2349–2361. 10.1093/jxb/err079 21430291

[B54] Tivoli B. Calonnec A. Richard B. Ney B. Andrivon D. (2013). Current knowledge on plant/canopy architectural traits that reduce the expression and development of epidemics. Eur. J. Plant Pathol. 135, 471–478. 10.1007/s10658-012-0066-6

[B55] Voorrips R. E. (2002). MapChart: software for the graphical presentation of linkage maps and QTLs. J. Heredity 93, 77–78. 10.1093/jhered/93.1.77 12011185

[B56] Weeden N. F. Moffet M. (2002). Identification of genes affecting root mass and root/shoot ratio in a JI 1794 x 'Slow' RIL population. Pisum Genet. 34, 28–31.

[B57] Wicker E. Rouxel F. (2001). Specific behaviour of French *Aphanomyces euteiches* drechs. Populations for virulence and aggressiveness on pea, related to isolates from Europe, America and New Zealand. Eur. J. Plant Pathol. 107, 919–929. 10.1023/a:1013171217610

