# Supplementary figures and images for: Comparative Genome-Wide-Association Mapping Identifies Common Loci Controlling Root System Architecture and Resistance to Aphanomyces euteiches in Pea
Source: Front Plant Sci. 2018 Jan 5;8:2195. doi: 10.3389/fpls.2017.02195 (PMC5761208; doi:10.3389/fpls.2017.02195)

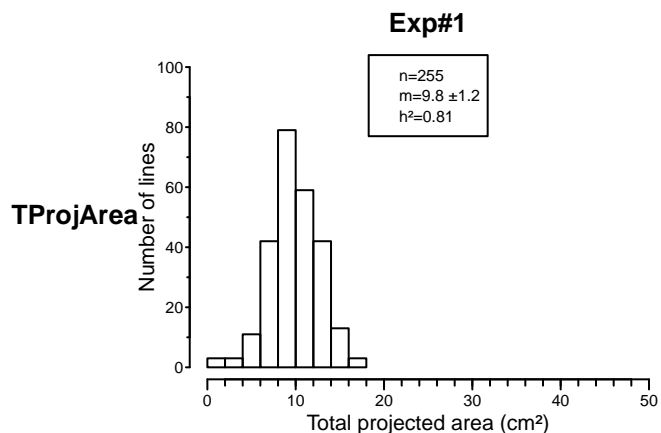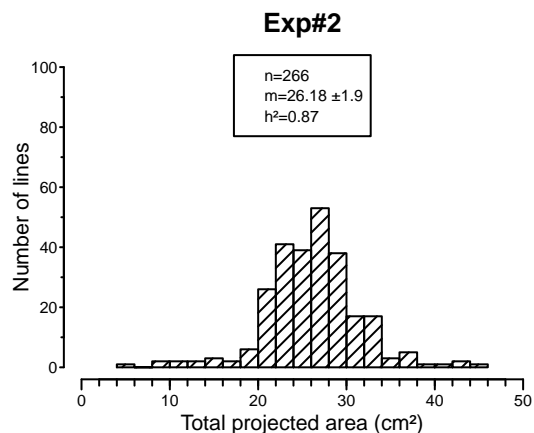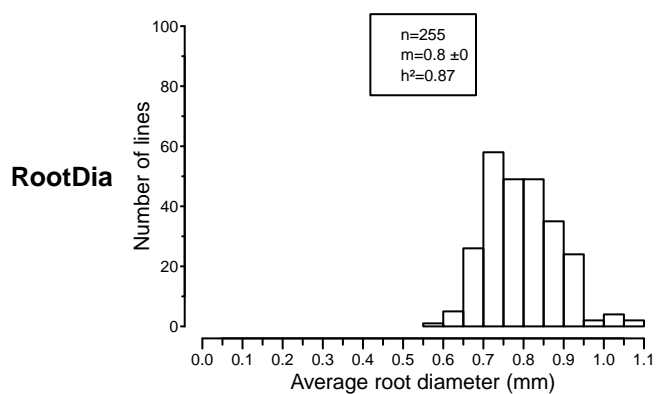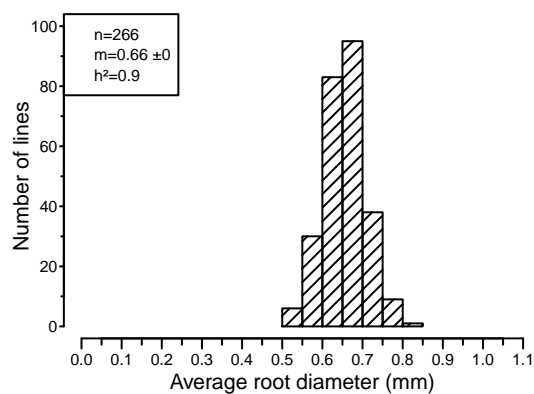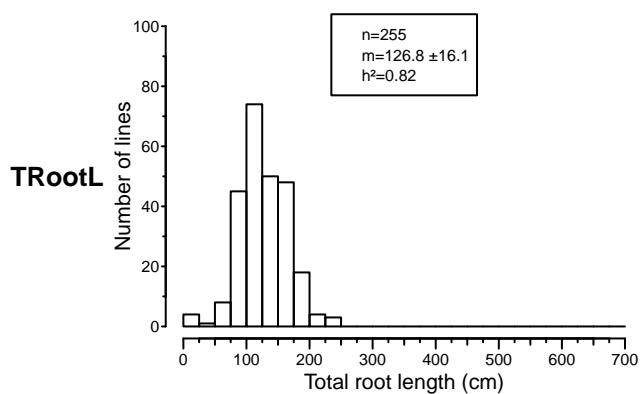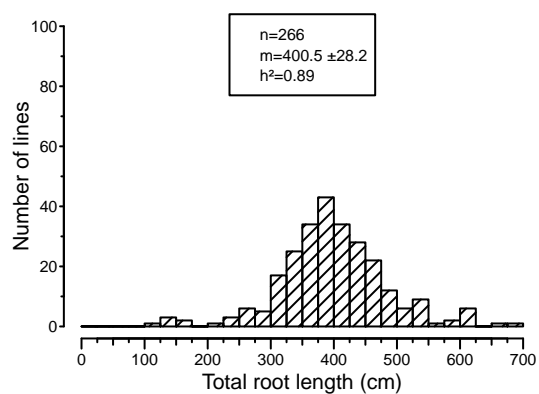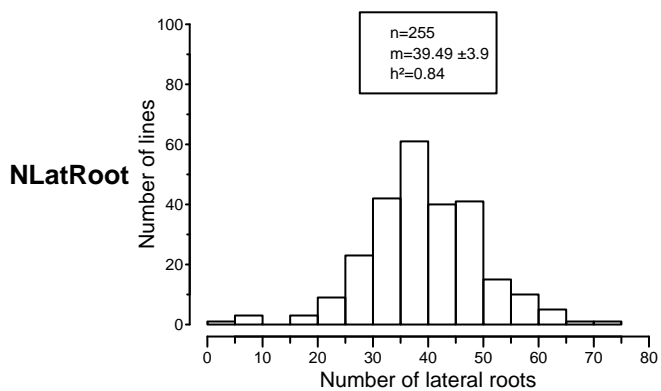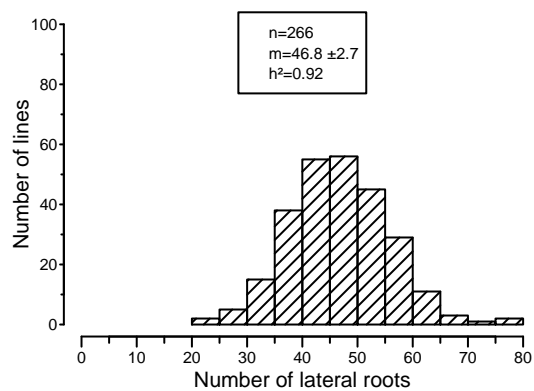

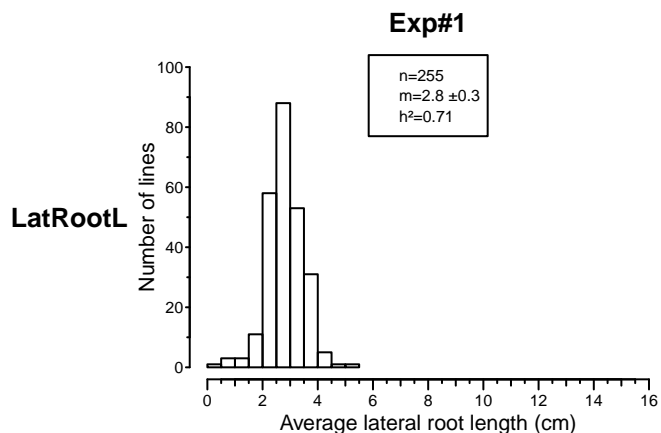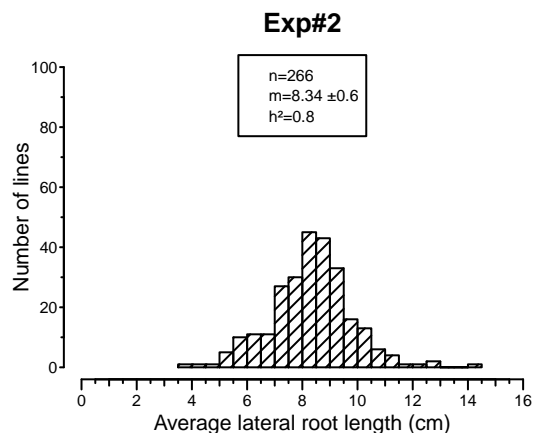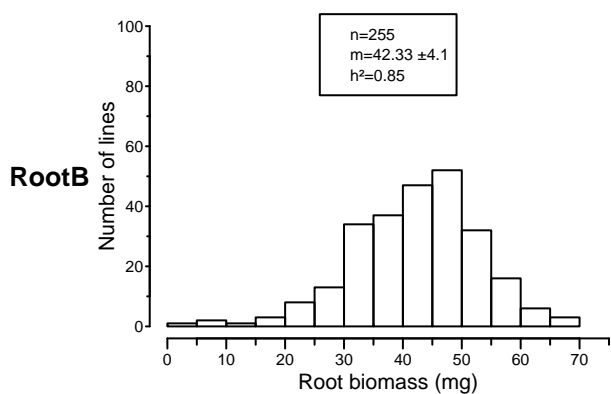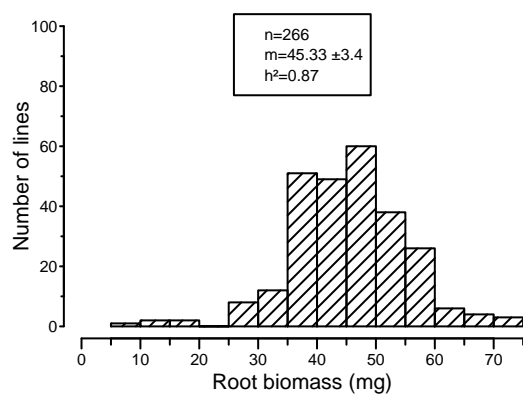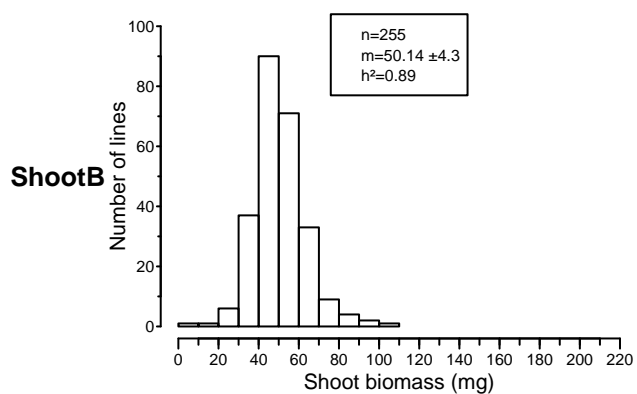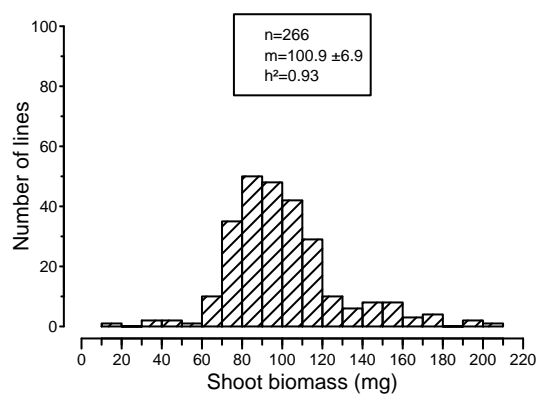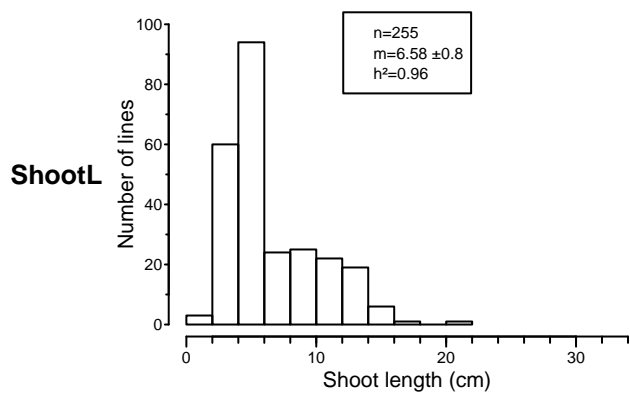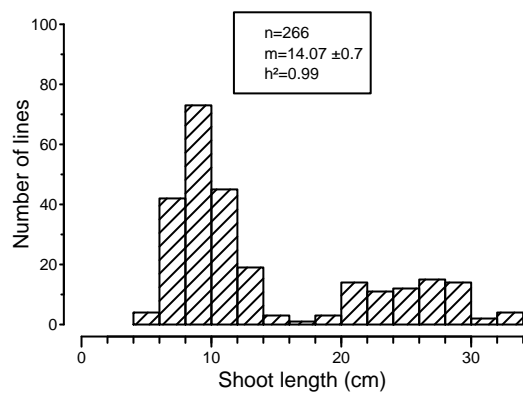

**TB**

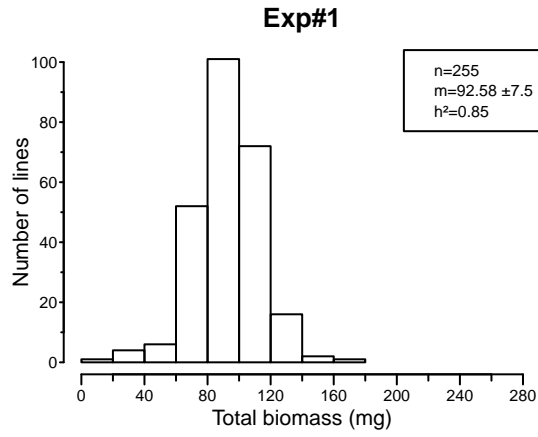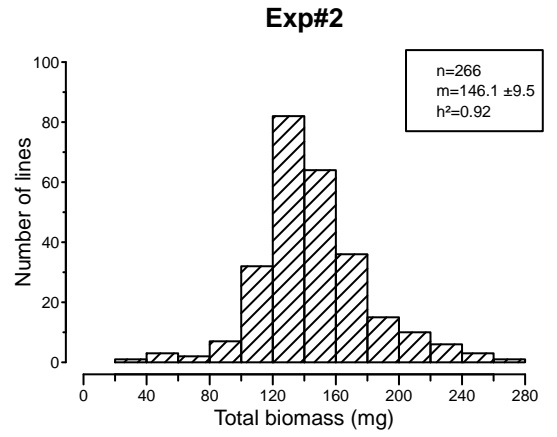

**RootB:TB**

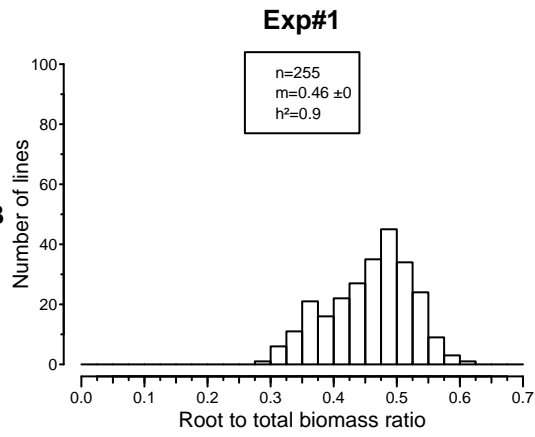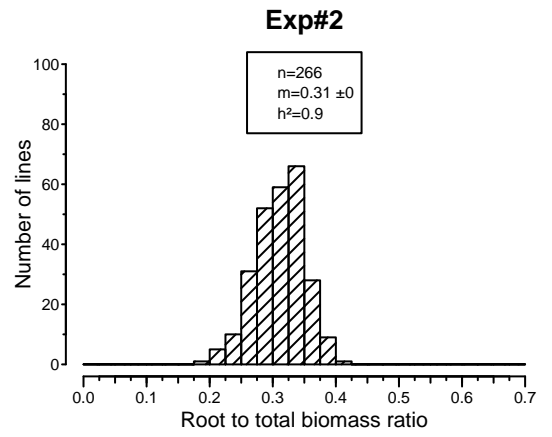

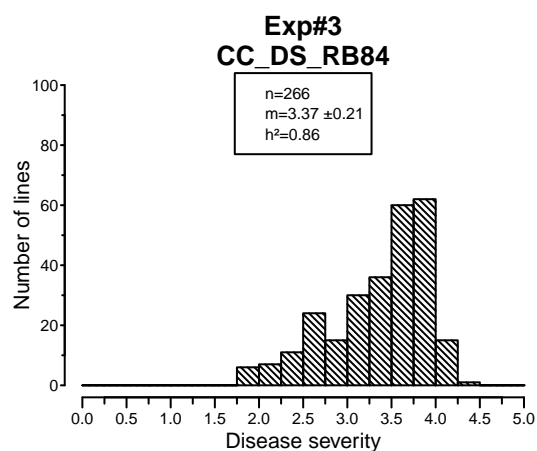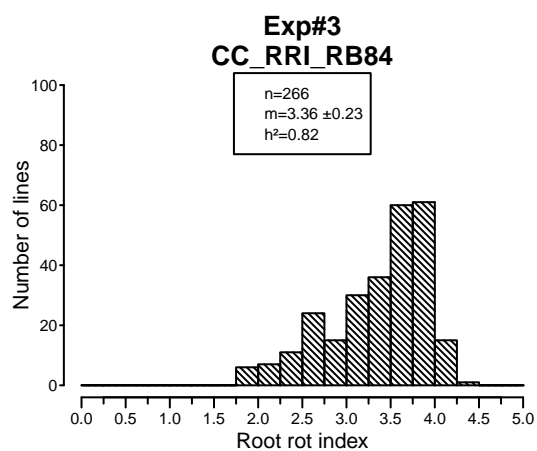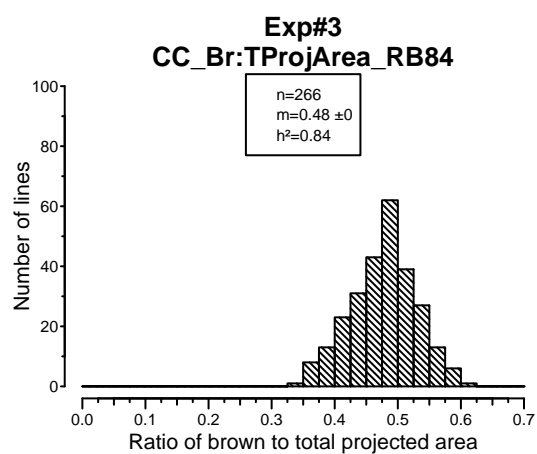

Supplement: Supplementary Figure 1 — Frequency distribution of least square means obtained for root, shoot and overall plant architecture variables, and for Aphanomyces root rot resistance in the 266-pea-line collection. Least square means were obtained from analysis of variance for six root system architecture variables (total projected area, average root diameter, total root length, number of lateral roots, average length of lateral roots, root biomass, coded as TProjArea, RootDia, TRootL, NLatRoot, LatRootL, RootB, respectively), two shoot architecture variables (shoot biomass and shoot length, coded as ShootB and ShootL, respectively), two overall plant architecture variables (total biomass and root to total biomass ratio, coded as TB and RootB:TB, respectively) and three Aphanomyces root rot resistance variables (disease severity, root rot index and percentage of brown projected area, coded DS, RRI and Br:TProjArea, respectively). Architectural traits were assessed on 8 day-old plants in a greenhouse (Exp#1) and 14 day-old plants in a climate controlled chamber (Exp#2). Aphanomyces resistance traits were assessed on 14 day-old plants inoculated at the 7th day with a reference strain of A. euteiches (RB84) in a climate controlled chamber (Exp#3). n: total number of pea lines assessed; m: mean ± standard deviation of the 266-pea-line collection; h2: mean-based heritability. [file Image1.PDF]

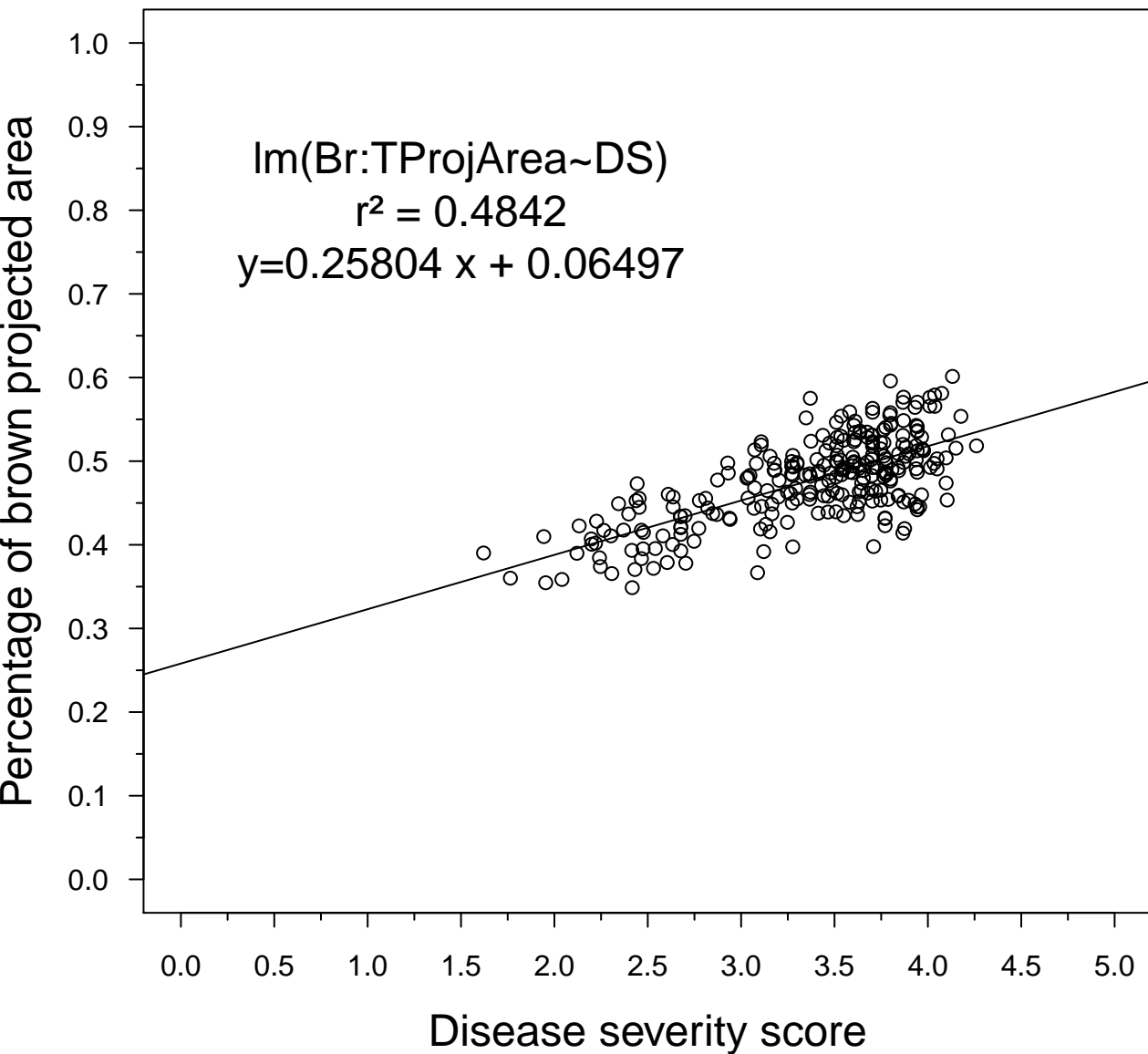

Supplement: Supplementary Figure 2 — Regression line between disease severity score and percentage of brown projected area measured in Exp#3. LSMeans of disease severity (DS) score was plotted against LSMeans of percentage of brown projected area (Br:TProjArea). Function of the regression line (y = 0.25804x + 0.06497) was calculated through the linear model Br:TProjArea DS. The regression coefficient r2 translates the relevance of the model. [file Image2.PDF]

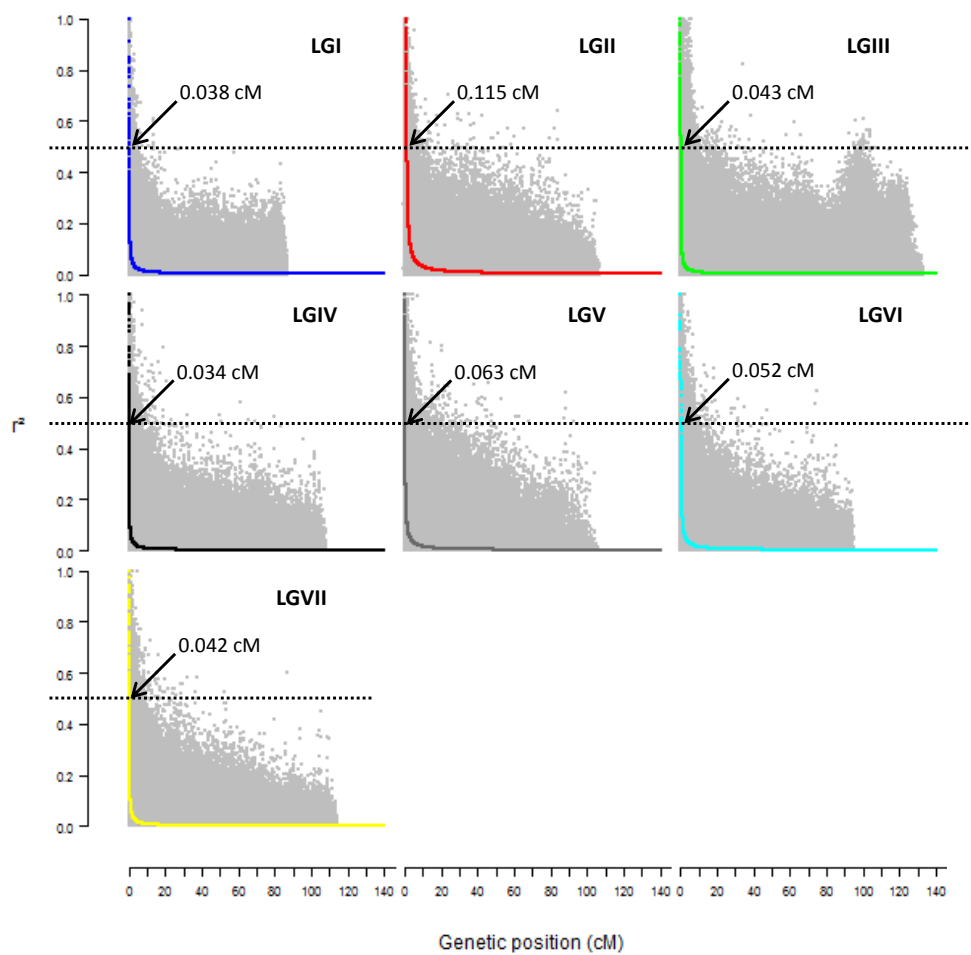

Supplement: Supplementary Figure 3 — Linkage disequilibrium (LD) decay in the 266-pea-line collection. Colored curves represent the estimated LD decay for each linkage group (LG). Dashed vertical lines represent the LD threshold (maximum r2/2) and arrows the LD decay rate, as the estimated genetic distance (cM) to reach this LD threshold on each LG. [file Image3.PDF]
