# Supplementary material for: Comparative Genome-Wide-Association Mapping Identifies Common Loci Controlling Root System Architecture and Resistance to Aphanomyces euteiches in Pea
Source: Front Plant Sci. 2018 Jan 5;8:2195. doi: 10.3389/fpls.2017.02195 (PMC5761208; doi:10.3389/fpls.2017.02195)
Supplement: Supplementary Figure 5 — Comparative genetic map of genome-wide association (GWA) and previously detected linkage quantitative trait loci (QTL) for resistance to A. euteiches and plant architecture on LGI to VI. The comparative genetic map was constructed from the projection of the 953 markers from Boutet et al. (2016) onto the consensus THMap from Desgroux et al. (2016). Linkage groups (LG) I to VI are shown (see Figure 3 for LGVII). LG size is indicated in cM Haldane. Shoot architecture-, root architecture-, overall plant architecture- and resistance- associated markers and QTL are indicated in green, orange, blue and red, respectively. To the right of each LG: Confidence intervals (CIs) around significant resistance-associated markers, based on linkage disequilibrium (LD) value r2 > 0.2, identified in this study by GWA and name of the trait are indicated (GWAS Exp#3); CIs around significant resistance-associated markers, identified in controlled conditions by GWA in Desgroux et al. (2016); Projected Meta-QTL (MQTL) and QTL (Ae-Ps QTL) described in Hamon et al. (2011) and Hamon et al. (2013), hatched bars represent Meta-QTL, while blank bars represent initial QTL before meta-analysis. The main Aphanomyces root rot resistance QTL and Meta-QTL names are in bold italic. To the left of each LG: Genomic positions of cloned pea genes are indicated in gray; CIs around plant architecture associated markers identified in this study (GWAS Exp#1 and GWAS Exp#2); Projected QTL for root, shoot and plant architecture traits described in Bourion et al. (2010). [file Image5.PDF]

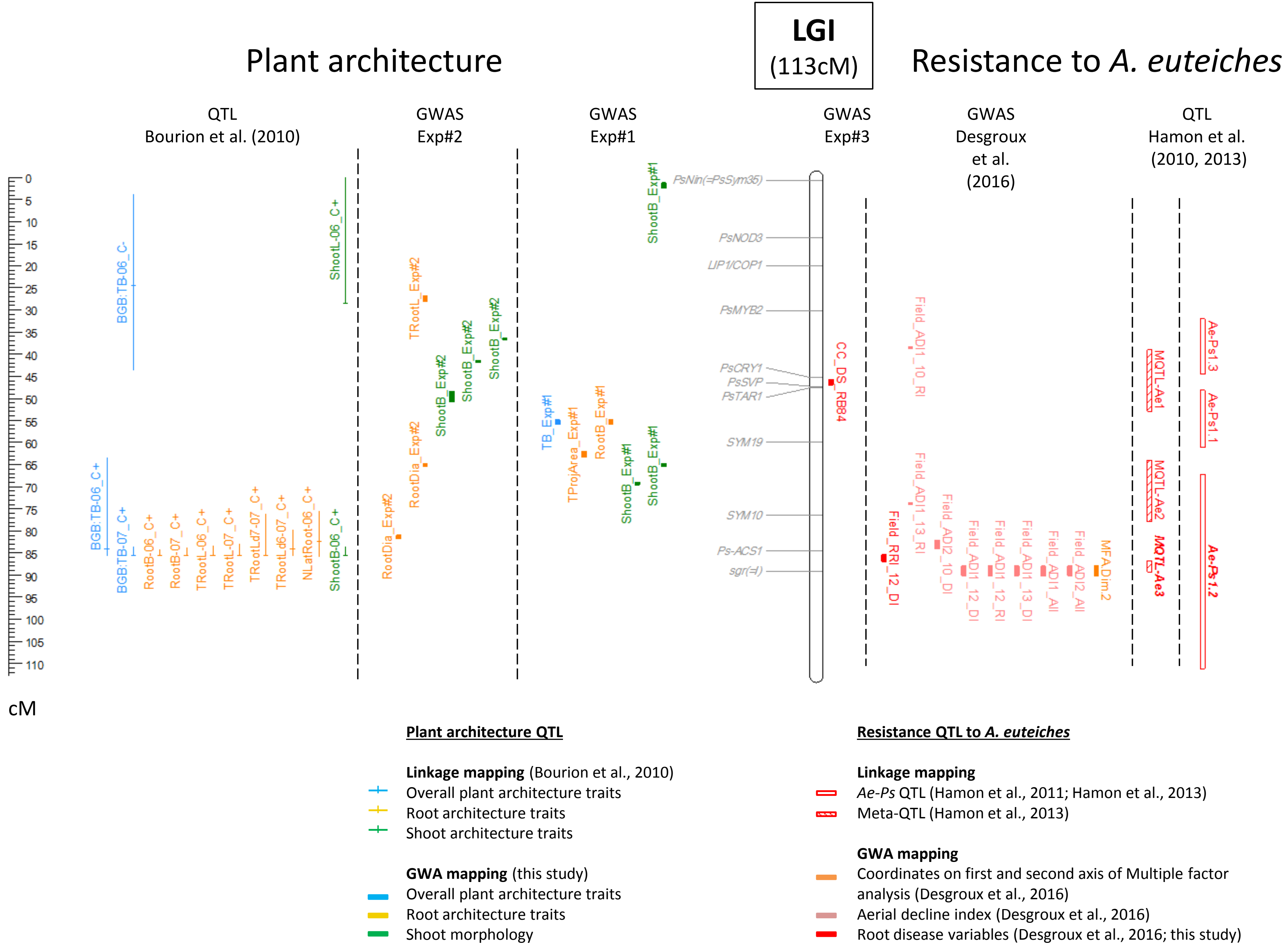
LGI
(113cM)
Plant architecture
Resistance to A. euteiches
QTL
Bourion et al. (2010)
GWAS
Exp#2
GWAS
Exp#1
GWAS
Exp#3
GWAS
Desgroux
et al.
(2016)
QTL
Hamon et al.
(2010, 2013)
cM
BGB:TB-06_C-
ShootL-06_C+
BGB:TB-06_C+
BGB:TB-07_C+
RootB-06_C+
RootB-07_C+
TRootL-06_C+
TRootL-07_C+
TRootLd7-07_C+
TRootLd6-07_C+
NLatRoot-06_C+
ShootB-06_C+
TRootL_Exp#2
ShootB_Exp#2
RootDia_Exp#2
TB_Exp#1
TProjArea_Exp#1
RootB_Exp#1
ShootB_Exp#1
PsNin(=PsSym35)
PsNOD3
LIP1/COP1
PsMYB2
PsCRY1
PsSVP
PsTAR1
SYM19
SYM10
Ps-ACS1
sgr(=I)
CC_DS_RB84
Field_ADI1_10_RI
Field_ADI1_13_RI
Field_RRI_12_DI
Field_ADI2_10_DI
Field_ADI1_12_DI
Field_ADI1_12_RI
Field_ADI1_13_DI
Field_ADI1_All
Field_ADI2_All
MFA.Dim.2
MQTL-Ae1
MQTL-Ae2
MQTL-Ae3
Ae-Ps1.3
Ae-Ps1.1
Ae-Ps1.2
Plant architecture QTL
Linkage mapping (Bourion et al., 2010)
Overall plant architecture traits
Root architecture traits
Shoot architecture traits
GWA mapping (this study)
Overall plant architecture traits
Root architecture traits
Shoot morphology
Resistance QTL to A. euteiches
Linkage mapping
Ae-Ps QTL (Hamon et al., 2011; Hamon et al., 2013)
Meta-QTL (Hamon et al., 2013)
GWA mapping
Coordinates on first and second axis of Multiple factor analysis (Desgroux et al., 2016)
Aerial decline index (Desgroux et al., 2016)
Root disease variables (Desgroux et al., 2016; this study)

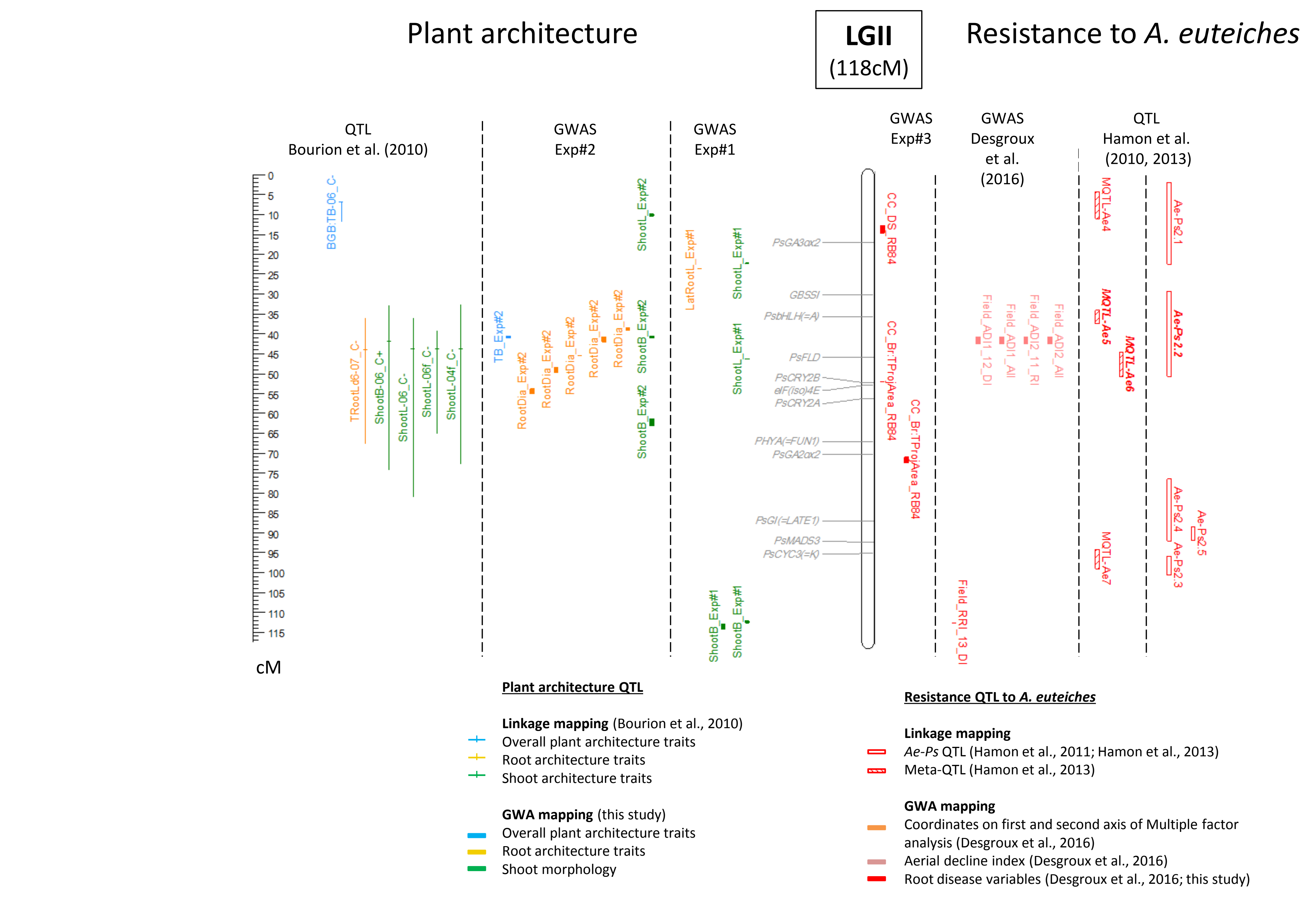
Plant architecture
LGII
(118cM)
Resistance to A. euteiches
QTL
Bourion et al. (2010)
GWAS
Exp#2
GWAS
Exp#1
GWAS
Exp#3
GWAS
Desgroux
et al.
(2016)
QTL
Hamon et al.
(2010, 2013)
cM
BGB:TB-06_C-
TRootLd6-07_C-
ShootB-06_C+
ShootL-06_C-
ShootL-06f_C-
ShootL-04f_C-
TB_Exp#2
RootDia_Exp#2
ShootL_Exp#2
ShootB_Exp#2
LatRootL_Exp#1
ShootL_Exp#1
ShootB_Exp#1
PsGA3ox2
GBSSI
PsbHLH(=A)
PsFLD
PsCRY2B
eIF(iso)4E
PsCRY2A
PHYA(=FUN1)
PsGA2ox2
PsGI(=LATE1)
PsMADS3
PsCYC3(=K)
CC_DS_RB84
CC_Br:TProjArea_RB84
Field_ADI1_12_DI
Field_ADI1_All
Field_ADI2_11_RI
Field_ADI2_All
Field_RRI_13_DI
MQTL-Ae4
MQTL-Ae5
MQTL-Ae6
MQTL-Ae7
Ae-Ps2.1
Ae-Ps2.2
Ae-Ps2.4
Ae-Ps2.5
Ae-Ps2.3
Plant architecture QTL
Linkage mapping (Bourion et al., 2010)
Overall plant architecture traits
Root architecture traits
Shoot architecture traits
GWA mapping (this study)
Overall plant architecture traits
Root architecture traits
Shoot morphology
Resistance QTL to A. euteiches
Linkage mapping
Ae-Ps QTL (Hamon et al., 2011; Hamon et al., 2013)
Meta-QTL (Hamon et al., 2013)
GWA mapping
Coordinates on first and second axis of Multiple factor analysis (Desgroux et al., 2016)
Aerial decline index (Desgroux et al., 2016)
Root disease variables (Desgroux et al., 2016; this study)

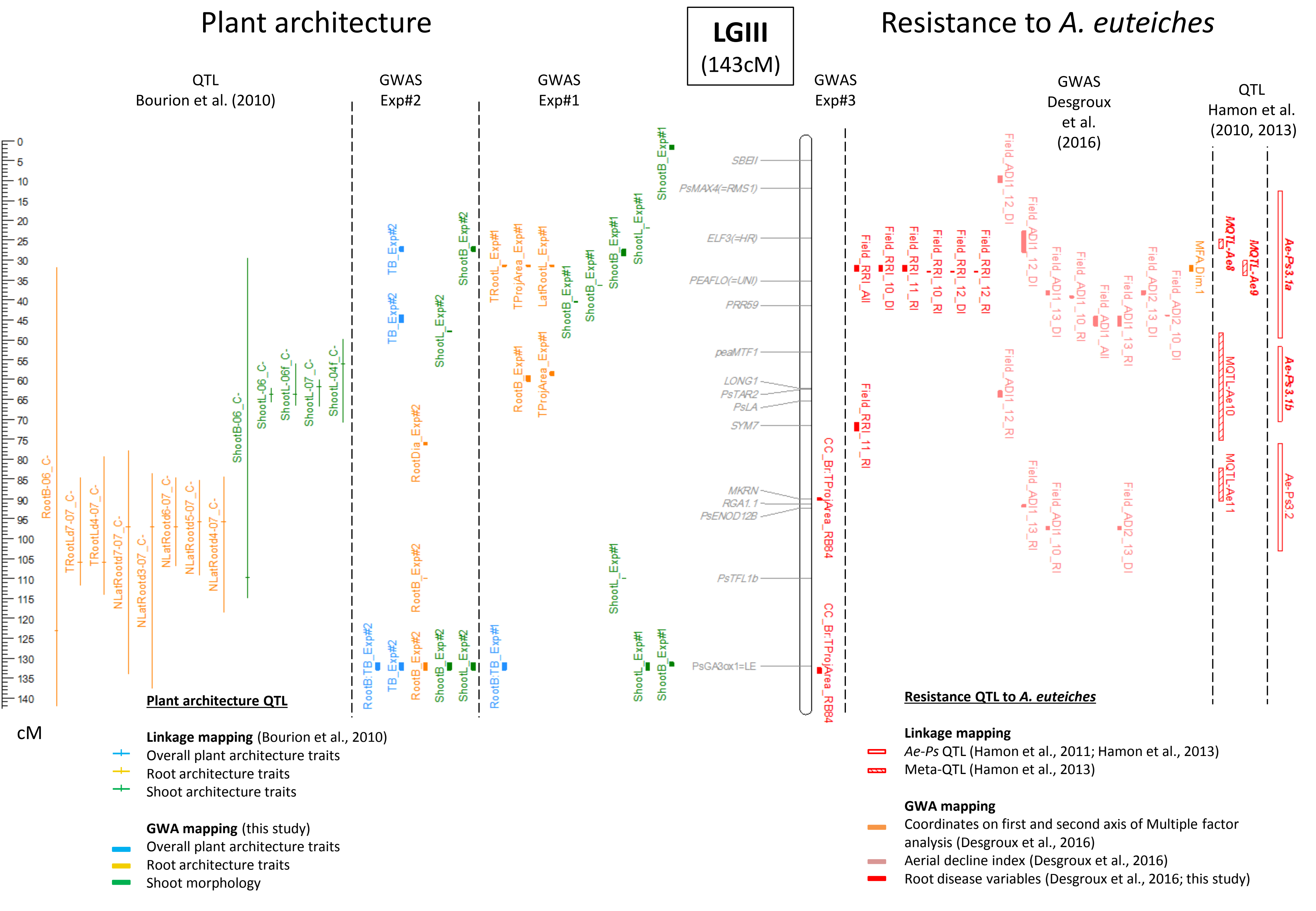

Plant architecture
LGIII
(143cM)
Resistance to A. euteiches
QTL
Bourion et al. (2010)
GWAS
Exp#2
GWAS
Exp#1
GWAS
Exp#3
GWAS
Desgroux
et al.
(2016)
QTL
Hamon et al.
(2010, 2013)
cM
SBEI
PsMAX4(=RMS1)
ELF3(=HR)
PEAFLO(=UNI)
PRR59
peaMTF1
LONG1
PsTAR2
PsLA
SYM7
MKRN
RGA1.1
PsENOD12B
PsTFL1b
PsGA3ox1=LE
MQTL-Ae8
MQTL-Ae9
MQTL-Ae10
MQTL-Ae11
Ae-Ps3.1a
Ae-Ps3.1b
Ae-Ps3.2
MFA_Dim.1
CC_Br.TProjArea_RB84
Plant architecture QTL
Linkage mapping (Bourion et al., 2010)
Overall plant architecture traits
Root architecture traits
Shoot architecture traits
GWA mapping (this study)
Overall plant architecture traits
Root architecture traits
Shoot morphology
Resistance QTL to A. euteiches
Linkage mapping
Ae-Ps QTL (Hamon et al., 2011; Hamon et al., 2013)
Meta-QTL (Hamon et al., 2013)
GWA mapping
Coordinates on first and second axis of Multiple factor analysis (Desgroux et al., 2016)
Aerial decline index (Desgroux et al., 2016)
Root disease variables (Desgroux et al., 2016; this study)

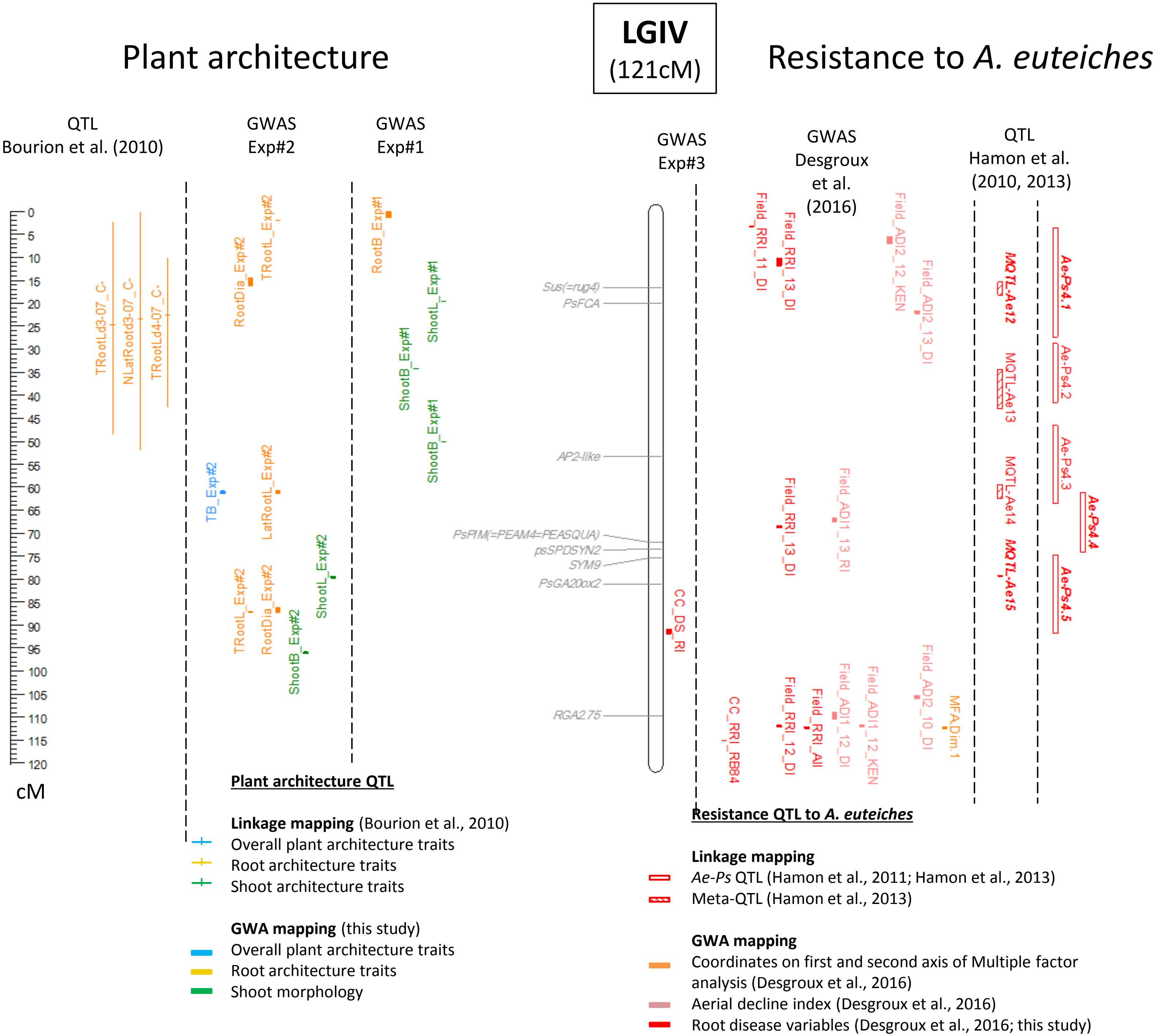

LGIV
(121cM)
Plant architecture
Resistance to A. euteiches
QTL
Bourion et al. (2010)
GWAS
Exp#2
GWAS
Exp#1
GWAS
Exp#3
GWAS
Desgroux
et al.
(2016)
QTL
Hamon et al.
(2010, 2013)
cM
TRootLd3-07_C-
NLatRootd3-07_C-
TRootLd4-07_C-
RootDia_Exp#2
TRootL_Exp#2
TB_Exp#2
LatRootL_Exp#2
RootDia_Exp#2
ShootB_Exp#2
ShootL_Exp#2
RootB_Exp#1
ShootL_Exp#1
ShootB_Exp#1
Sus(=rug4)
PsFCA
AP2-like
PsPIM(=PEAM4=PEASQUA)
psSPDSYN2
SYM9
PsGA20ox2
RGA2.75
CC_DS_RI
Field_RRI_11_DI
Field_RRI_13_DI
Field_ADI2_12_KEN
Field_ADI2_13_DI
Field_ADI1_13_RI
CC_RRI_RB84
Field_RRI_12_DI
Field_RRI_All
Field_ADI1_12_DI
Field_ADI1_12_KEN
Field_ADI2_10_DI
MFA.Dim.1
MQTL-Ae12
MQTL-Ae13
MQTL-Ae14
MQTL-Ae15
Ae-Ps4.1
Ae-Ps4.2
Ae-Ps4.3
Ae-Ps4.4
Ae-Ps4.5
Plant architecture QTL
Linkage mapping (Bourion et al., 2010)
Overall plant architecture traits
Root architecture traits
Shoot architecture traits
GWA mapping (this study)
Overall plant architecture traits
Root architecture traits
Shoot morphology
Resistance QTL to A. euteiches
Linkage mapping
Ae-Ps QTL (Hamon et al., 2011; Hamon et al., 2013)
Meta-QTL (Hamon et al., 2013)
GWA mapping
Coordinates on first and second axis of Multiple factor analysis (Desgroux et al., 2016)
Aerial decline index (Desgroux et al., 2016)
Root disease variables (Desgroux et al., 2016; this study)

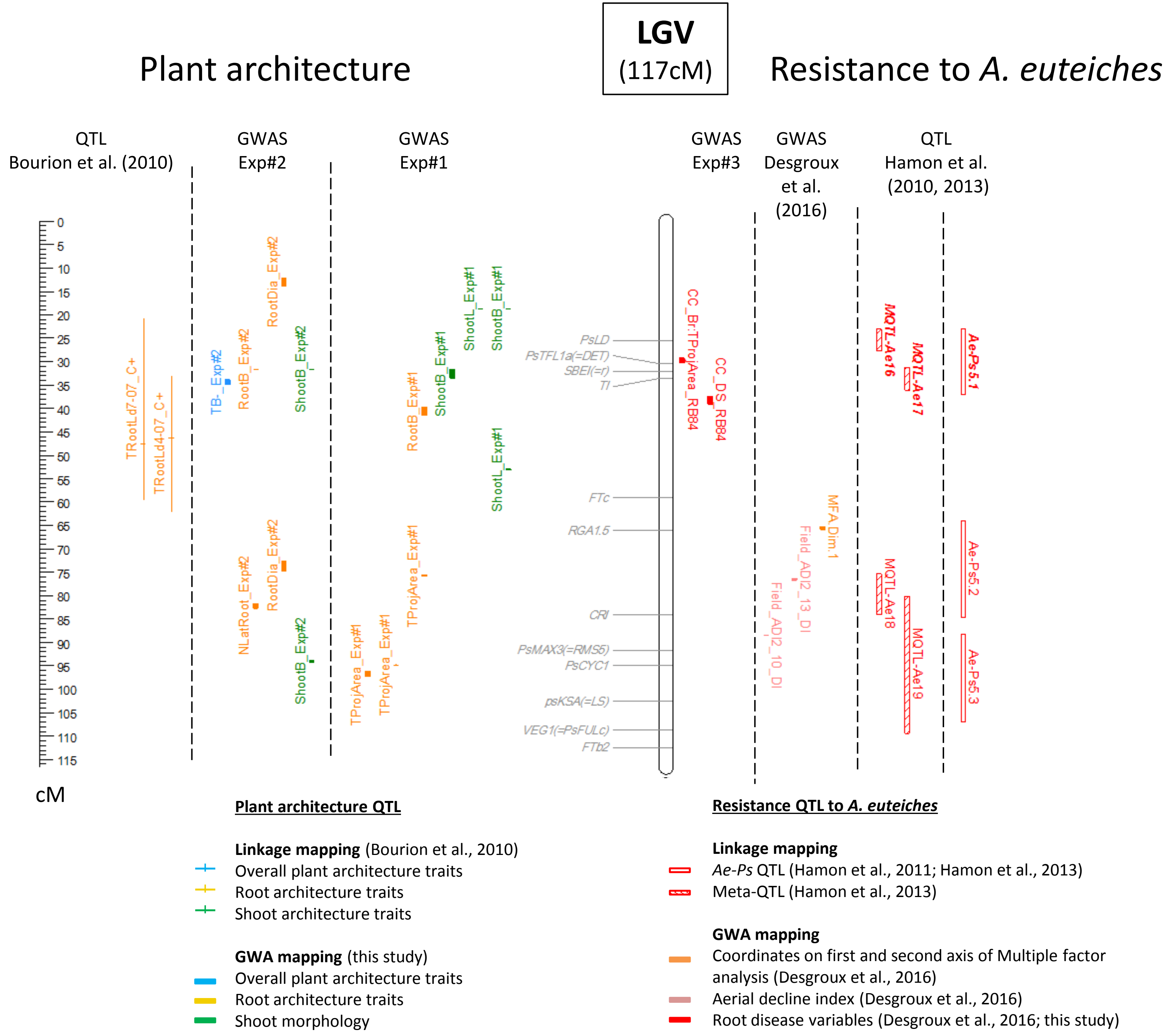

LGV
(117cM)
Plant architecture
Resistance to A. euteiches
QTL
Bourion et al. (2010)
GWAS
Exp#2
GWAS
Exp#1
GWAS
Exp#3
GWAS
Desgroux
et al.
(2016)
QTL
Hamon et al.
(2010, 2013)
cM
TRootLd7-07_C+
TRootLd4-07_C+
TB-_Exp#2
RootB_Exp#2
RootDia_Exp#2
ShootB_Exp#2
NLatRoot_Exp#2
RootDia_Exp#2
ShootB_Exp#2
RootB_Exp#1
ShootB_Exp#1
ShootL_Exp#1
ShootB_Exp#1
ShootL_Exp#1
TProjArea_Exp#1
TProjArea_Exp#1
TProjArea_Exp#1
PsLD
PsTFL1a(=DET)
SBEI(=r)
Tl
FTc
RGA1.5
CRI
PsMAX3(=RMS5)
PsCYC1
psKSA(=LS)
VEG1(=PsFULc)
FTb2
CC_Br:TProjArea_RB84
CC_DS_RB84
MFA_Dim.1
Field_ADI2_13_DI
Field_ADI2_10_DI
MQTL-Ae16
MQTL-Ae17
MQTL-Ae18
MQTL-Ae19
Ae-Ps5.1
Ae-Ps5.2
Ae-Ps5.3
Plant architecture QTL
Linkage mapping (Bourion et al., 2010)
Overall plant architecture traits
Root architecture traits
Shoot architecture traits
GWA mapping (this study)
Overall plant architecture traits
Root architecture traits
Shoot morphology
Resistance QTL to A. euteiches
Linkage mapping
Ae-Ps QTL (Hamon et al., 2011; Hamon et al., 2013)
Meta-QTL (Hamon et al., 2013)
GWA mapping
Coordinates on first and second axis of Multiple factor analysis (Desgroux et al., 2016)
Aerial decline index (Desgroux et al., 2016)
Root disease variables (Desgroux et al., 2016; this study)

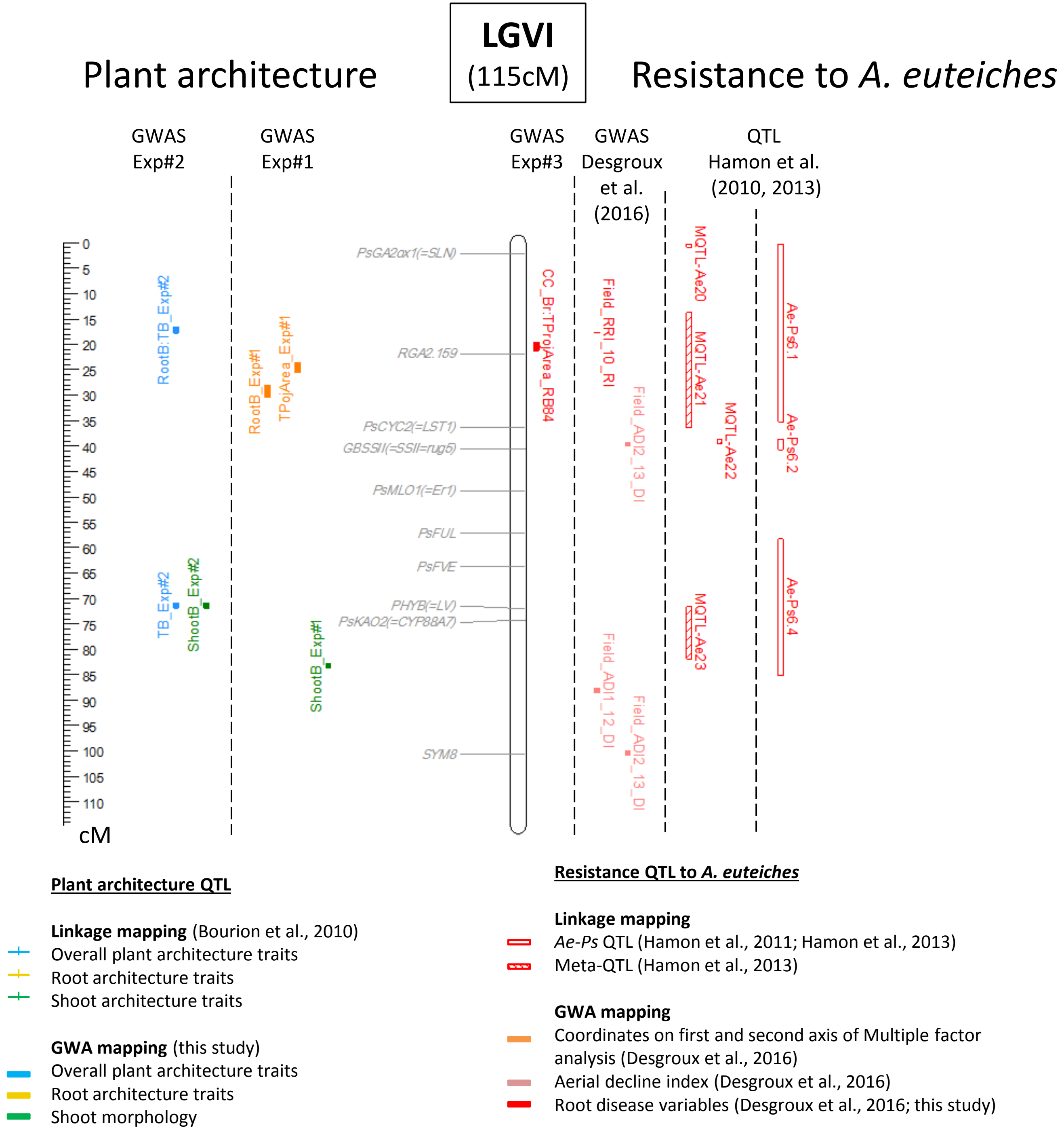
LGVI
(115cM)
Plant architecture
Resistance to A. euteiches
GWAS Exp#2
GWAS Exp#1
GWAS Exp#3
GWAS Desgroux et al. (2016)
QTL Hamon et al. (2010, 2013)
cM
RootB:TB_Exp#2
TB_Exp#2
ShootB_Exp#2
RootB_Exp#1
TPojArea_Exp#1
ShootB_Exp#1
PsGA2ox1(=SLN)
RGA2.159
PsCYC2(=LST1)
GBSSII(=SSII=rug5)
PsMLO1(=Er1)
PsFUL
PsFVE
PHYB(=LV)
PsKAO2(=CYP88A7)
SYM8
CC_Br:TProjArea_RB84
Field_RRI_10_RI
Field_ADI2_13_DI
Field_ADI1_12_DI
Field_ADI2_13_DI
MQTL-Ae20
MQTL-Ae21
MQTL-Ae22
MQTL-Ae23
Ae-Ps6.1
Ae-Ps6.2
Ae-Ps6.4
Plant architecture QTL
Linkage mapping (Bourion et al., 2010)
Overall plant architecture traits
Root architecture traits
Shoot architecture traits
GWA mapping (this study)
Overall plant architecture traits
Root architecture traits
Shoot morphology
Resistance QTL to A. euteiches
Linkage mapping
Ae-Ps QTL (Hamon et al., 2011; Hamon et al., 2013)
Meta-QTL (Hamon et al., 2013)
GWA mapping
Coordinates on first and second axis of Multiple factor analysis (Desgroux et al., 2016)
Aerial decline index (Desgroux et al., 2016)
Root disease variables (Desgroux et al., 2016; this study)
