# Supplementary material for: Comparative Genome-Wide-Association Mapping Identifies Common Loci Controlling Root System Architecture and Resistance to Aphanomyces euteiches in Pea
Source: Front Plant Sci. 2018 Jan 5;8:2195. doi: 10.3389/fpls.2017.02195 (PMC5761208; doi:10.3389/fpls.2017.02195)
Supplement: Supplementary Figure 4 — Clustered heatmap of the Kinship matrix of the 266-pea-line collection.Kinship matrix from the GAPIT R package (Lipka et al., 2012) based on 2,937 SNP markers. Clustering is based on the UPGMA method. The color gradient represents the degree of relationship between two lines shown. Kinship matrix from the GAPIT R package (Lipka et al., 2012) based on 2,937 SNP markers. Clustering is based on the UPGMA method. The color gradient represents the degree of relationship between two lines shown. [file Image4.PDF]

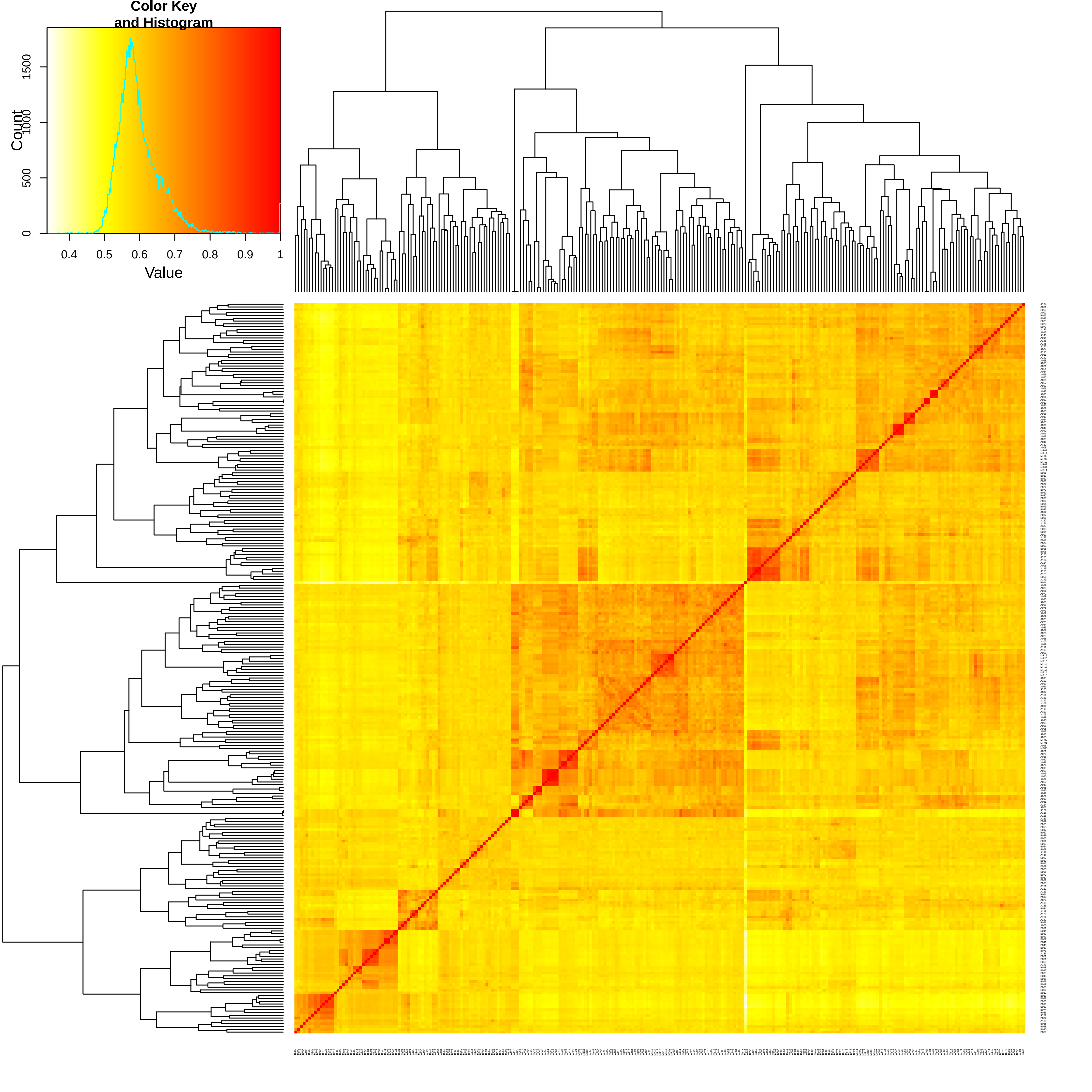
Color Key
and Histogram
Count
Value
0
500
1000
1500
0.4
0.5
0.6
0.7
0.8
0.9
1
